# Patient and General Population Preferences Regarding the Benefits and Harms of Treatment for Metastatic Prostate Cancer: A Discrete Choice Experiment

**DOI:** 10.1016/j.euros.2023.03.001

**Published:** 2023-03-22

**Authors:** Dominik Menges, Michela C. Piatti, Aurelius Omlin, Richard Cathomas, Daniel Benamran, Stefanie Fischer, Christophe Iselin, Marc Küng, Anja Lorch, Lukas Prause, Christian Rothermundt, Alix O'Meara Stern, Deborah Zihler, Max Lippuner, Julia Braun, Thomas Cerny, Milo A. Puhan

**Affiliations:** a Epidemiology, Biostatistics and Prevention Institute, University of Zurich, Zurich, Switzerland; b Department of Medical Oncology and Hematology, Kantonsspital St. Gallen, St. Gallen, Switzerland; c Onkozentrum Zürich, Zurich, Switzerland; d Division of Oncology/Hematology, Kantonsspital Graubünden, Chur, Switzerland; e Department of Urology, Hôpitaux Universitaires Genève, Geneva, Switzerland; f Department of Oncology, Hôpital Cantonal Fribourg, Fribourg, Switzerland; g Department of Medical Oncology and Hematology, University Hospital Zurich, Zurich, Switzerland; h Department of Urology, Kantonsspital Aarau, Aarau, Switzerland; i Department of Oncology, Réseau Hospitalier Neuchâtelois, Neuchâtel, Switzerland; j Department of Oncology, Hematology and Transfusion Medicine, Kantonsspital Aarau, Aarau, Switzerland; k Europa Uomo Switzerland, Ehrendingen, Switzerland; l Foundation Board, Cancer Research Switzerland, Bern, Switzerland; m Human Medicines Expert Committee, Swissmedic, Bern, Switzerland

**Keywords:** Adverse effects, Benefit, Benefit-harm assessment, Discrete choice experiment, Harm, Metastasis, Patient-centered care, Patient preferences, Prostate cancer, Survival, Treatment

## Abstract

**Background:**

Patient preferences for treatment outcomes are important to guide decision-making in clinical practice, but little is known about the preferences of patients with metastatic hormone-sensitive prostate cancer (mHSPC).

**Objective:**

To evaluate patient preferences regarding the attributed benefits and harms of systemic treatments for mHSPC and preference heterogeneity between individuals and specific subgroups.

**Design, setting, and participants:**

We conducted an online discrete choice experiment (DCE) preference survey among 77 patients with metastatic prostate cancer (mPC) and 311 men from the general population in Switzerland between November 2021 and August 2022.

**Outcome measurements and statistical analysis:**

We evaluated preferences and preference heterogeneity related to survival benefits and treatment-related adverse effects using mixed multinomial logit models and estimated the maximum survival time participants were willing to trade to avert specific adverse effects. We further assessed characteristics associated with different preference patterns via subgroup and latent class analyses.

**Results and limitations:**

Patients with mPC showed an overall stronger preference for survival benefits in comparison to men from the general population (*p* = 0.004), with substantial preference heterogeneity between individuals within the two samples (both *p* < 0.001). There was no evidence of differences in preferences for men aged 45–65 yr versus ≥65 yr, patients with mPC in different disease stages or with different adverse effect experiences, or general population participants with and without experiences with cancer. Latent class analyses suggested the presence of two groups strongly preferring either survival or the absence of adverse effects, with no specific characteristic clearly associated with belonging to either group. Potential biases due to participant selection, cognitive burden, and hypothetical choice scenarios may limit the study results.

**Conclusions:**

Given the relevant heterogeneity in participant preferences regarding the benefits and harms of treatment for mHSPC, patient preferences should be explicitly discussed during decision-making in clinical practice and reflected in clinical practice guidelines and regulatory assessment regarding treatment for mHSPC.

**Patient summary:**

We examined the preferences (values and perceptions) of patients and men from the general population regarding the benefits and harms of treatment for metastatic prostate cancer. There were large differences between men in how they balanced the expected survival benefits and potential adverse effects. While some men strongly valued survival, others more strongly valued the absence of adverse effects. Therefore, it is important to discuss patient preferences in clinical practice.

## Take Home Message

There is substantial preference heterogeneity between patients regarding the expected survival benefits and potential adverse effects of systemic treatment for metastatic hormone-sensitive prostate cancer. Patients’ preferences regarding survival and treatment-related adverse effects should be explicitly discussed in clinical practice.

## 1 Introduction

Patient preferences are an important part of patient-centered care and there is growing interest in incorporating preference information in regulatory assessments of novel treatments [1], [2], [3], [4], [5], [6], [7]. In recent years, changes in the treatment landscape have vastly increased the number of treatment options for metastatic prostate cancer (mPC) [8], [9], [10], [11]. Various treatment options are available for metastatic hormone-sensitive prostate cancer (mHSPC) in addition to androgen deprivation therapy, including chemotherapy with docetaxel, second-generation hormonal therapy with abiraterone acetate, enzalutamide, or apalutamide, radiotherapy, and combined approaches [12], [13], [14], [15], [16]. However, this poses challenges in choosing a treatment on the basis of its expected benefits and potential harms. Since prostate cancer is the most frequent cancer among men [17], better knowledge about the treatment outcome–related preferences of patients with mPC is essential to optimally guide care and support these patients in clinical practice.

Preference studies are a well-established method for eliciting patient preferences [18], [19]. To date, only little such research has been conducted in mPC [20], [21]. Previous studies have shown that overall survival, progression-free survival, health-related quality of life (HRQoL), pain control, mode of administration, and the risk and severity of various potential adverse effects of treatment are important to patients with mPC [22], [23], [24], [25], [26], [27], [28], [29], [30]. Given the relevant variation between studies, it remains unclear which factors are most important for treatment decisions [20], [21]. Methodological discussions are ongoing on whether it is most appropriate to elicit preferences among individuals with or without experiences of the decision and its consequences, such as adverse effects during cancer treatment [2], [31]. In mPC, no study so far has addressed whether preferences differ between these groups [20], [21]. Furthermore, previous studies have not explicitly evaluated whether and how preferences vary between individual patients and patient subgroups [21]. However, such preference heterogeneity is highly important in clinical practice, since decision contexts with variable preferences are most likely to be preference-sensitive, warranting individualized treatment discussions [2], [32].

The aim of our study was to evaluate the preferences of patients with mPC and men from the general population regarding the attributed benefits and harms of systemic treatment for mHSPC. The main objectives were to elicit preference weights and estimate the trade-offs involved, compare preferences between the two populations, and evaluate preference heterogeneity between individuals and population subgroups.

## 2 Patients and methods

### 2.1 Study design

We conducted a cross-sectional preference survey between November 2021 and August 2022, following the good practice guidelines of the International Society For Pharmacoeconomics and Outcomes Research [19], [33], [34]. The study protocol was registered on the Open Science Framework platform [35]. The research project was evaluated by the Cantonal Ethics Committee of Zurich, Switzerland, and did not require ethical approval under the Swiss Human Research Act (BASEC Req-2020-00032). Further details on the methods are reported in the Supplementary material.

### 2.2 Participant recruitment

We enrolled two participant samples in this study, consisting of patients with mPC and men from the general population in Switzerland. Patients with mPC were recruited at seven sites by involved clinical experts during regular clinical consultations. While the target decision context was mHSPC, we considered all patients with mPC (mHSPC or metastatic castration-resistant prostate cancer [mCRPC]) who were undergoing or had previously undergone systemic treatment for mPC to be eligible, since we assumed that previous treatment experiences across different disease stages would have a similar influence regarding patients’ stated preferences in this study. Further eligibility criteria for the mPC group were age ≥18 yr and residence in Switzerland. Men from the general population were recruited via the respondent panel of a Swiss social and market research institute (LINK), stratified by age (45–64 yr and ≥65 yr) and language region. Eligible participants had to have no medical history of cancer, be aged ≥45 yr, and reside in Switzerland. All participants provided written (mPC group) or electronic (general population) consent and received a financial incentive for their participation.

### 2.3 Experimental design

We used a discrete choice experiment (DCE) approach, which is a method commonly used to quantitatively elicit preferences [18], [19], [33]. The DCE design was based on a qualitative exploration of patient preferences in mPC and pilot testing (Supplementary material). In brief, we conducted a systematic literature review of patient preferences to identify patient-relevant attributes of treatment for mPC [21]. We also conducted semi-structured phone interviews with 13 patients with mPC and six clinical experts (medical oncology, urology) to gather insights into the most important benefits and harms and factors determining the importance of adverse effects of treatments for mHSPC. Additional information sources consisted of a systematic literature review of treatments for mHSPC [14], European Medicines Agency and Swissmedic product labels for approved treatments in this context, and screening of the general literature on patient preferences. On the basis of the information retrieved, we mapped and selected patient-relevant attributes and attribute levels according to prespecified principles. The final DCE included seven attributes, defined as overall survival, diarrhea, fatigue, peripheral (sensory) neuropathy, fractures, ischemic heart disease, and rash, with three to four corresponding attribute levels each (duration of survival or levels of severity for adverse effects; Supplementary Table 2).

The DCE consisted of 16 individual choice tasks in which participants were asked to choose their preferred option from two hypothetical treatments with varying combinations of survival benefits and adverse effects. We determined the experimental design for the DCE using a Bayesian D-efficient design derived via a coordinate exchange algorithm implemented in the *idefix* (v1.0.3) package in *R* [36], [37]. We applied predetermined priors to produce 50 candidate sets of 15 choice tasks, from which we selected the DCE design that ensured a high design efficiency and attribute balance. We did not include a no-treatment (“opt out”) option as our aim was to estimate the trade-offs rather than determine real-life treatment choices. No blocking was applied to maximize the information gained on preference heterogeneity. We also included one choice task with a clearly dominant treatment option (higher benefit and lower harms; dominance test). The dominant choice task was fixed at the twelfth position, while the order of the remaining DCE tasks was randomized for each participant.

We performed pilot testing of the full questionnaire and study documentation in a sample of 12 patients with mPC and 20 men from the general population. Pilot test participants were asked to provide feedback regarding each part of the electronic survey, including the design, content, and wording of explanations, questions, and attribute descriptions in comment fields. We also collected structured feedback regarding the DCE tasks using targeted questions. On the basis of results from the pilot testing, we made minor changes to the wording of some questions and attribute descriptions, while the DCE design remained the same. We performed pilot testing in German, and the final questionnaire was subsequently translated into French and Italian.

### 2.4 Survey administration

The study was administered using an online survey platform. Patients with mPC had the additional option of completing a paper-based survey and requesting support via telephone. In addition to the DCE, the survey questionnaire included questions on sociodemographic characteristics, current health status (assessed via a visual analog scale [VAS]), and comorbidities. In addition, patients with mPC were asked for details regarding their initial diagnosis and metastasis, current and previous treatments, and any adverse effects of treatment they experienced. Men from the general population were surveyed using almost identical questionnaires, with specific questions on whether they had personal or professional experiences with cancer (ie, any personal experiences with prostate cancer or cancer more generally via affected relatives or friends, or professional experiences in treating or caring for individuals affected by cancer).

**Fig. 1:**
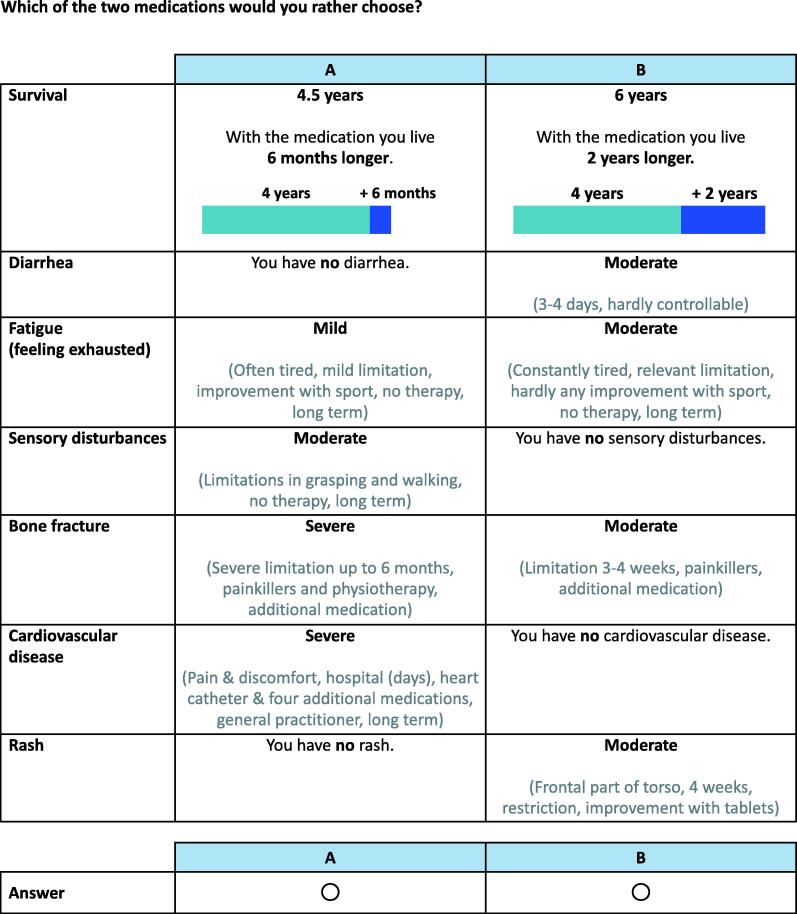
Example of a choice task from the discrete choice experiment regarding the benefits and harms of treatment for metastatic hormone-sensitive prostate cancer.

An example DCE choice task is provided in Fig. 1. The study documentation included a brief explanation of the aims of the study, a description of the (hypothetical) decision-making scenario, and an instruction on completing the choice tasks. We also presented participants with brief outcome descriptions for all attributes and attribute levels (accessible throughout the entire DCE), and included short cues given within all choice tasks for their reference. A visual representation was used for overall survival benefits, but not for the severity of adverse effects, since we wanted the outcome descriptions to drive participants’ choices rather than a visual representation of severity levels.

### 2.5 Statistical analysis

All participants providing at least partial data were included, while individuals providing no usable data in the DCE were excluded from the analysis. We categorized patients with mPC according to their disease stage (mHSPC or mCRPC) on the basis of their responses and information retrieved from their treating physicians. We performed a descriptive analysis of participant characteristics and assessed the internal validity of responses by analyzing the proportion of participants failing the dominance test, consistently choosing the same answer option, consistently responding on the basis of a single attribute, or completing the questionnaire in less than 15 min.

To derive preference weights for the different attribute levels, we applied multinomial logit models to the DCE choice data using dummy coding based on random utility theory [18], [34], [38], [39]. First, we explored the data using multinomial logit models with overall survival coded as either a categorical or continuous outcome. To evaluate preference heterogeneity and trade-offs between survival benefits and treatment adverse effects across individuals in the study, we then applied mixed multinomial logit models using overall survival (coded as a continuous outcome) as a random parameter in the primary analysis. Models were estimated on the basis of 100 Halton draws assuming a normal distribution for random parameters. We estimated separate models for the two study participant samples, as well as for the overall sample. Differences in preferences between participant samples and subgroups were evaluated using z-test statistics for coefficients derived from the separate models. We assessed and compared preference heterogeneity by evaluating the estimated standard deviations (SDs) for mean random parameter estimates. We then calculated the marginal rates of substitution to quantify the number of months of survival participants would trade against averting the harms by calculating the ratio of preference weights for adverse effects and a 1-yr increase in overall survival multiplied by 12.

We conducted several sensitivity analyses in which we excluded participants failing the internal validity assessments, estimated alternative models in which all attributes were included as random parameters, and used a 1:1 propensity score–matched subsample of study participants to evaluate differences between populations. We also conducted subgroup analyses to investigate whether there were differences in preferences between participants aged ≥65 yr and those aged <65 yr, between patients with mPC in different disease stages and with and without prior adverse effect experiences, and between men from the general population with and without personal or professional experiences with cancer. Finally, we conducted a prespecified experimental analysis using a latent class multinomial logit model to investigate whether there is evidence of the presence of two groups with different preferences. The hypothesis was that some individuals may strongly prefer survival and accept adverse effects, while others may more strongly prefer the absence of adverse effects (ie, higher HRQoL) and accept trade-offs regarding survival. We explored participant characteristics that may be associated with latent classes in a descriptive analysis and using multivariable logistic regression.

All statistical analyses were performed in *R* v4.1.1 using the *gmnl* v1.1-3.2 package [36], [40].

## 3 Results

### 3.1 Sample characteristics

We enrolled an overall sample of 388 individuals, composed of 77 patients with mPC and 311 men from the general population. The participation rate was 65.4% among eligible and invited patients with mPC and 63.1% among eligible men from the general population (Supplementary Fig. 2). Owing to limited patient enrolment, data from patients with mPC who participated in the pilot testing were included in this analysis (discussed in the Supplementary material).

Participant characteristics in the two populations differed: patients with mPC were older overall (median 73 yr vs 64 yr) and were more likely to be retired (77.6% vs 52.4%), reported lower general health status (median VAS score 75 vs 85), and more frequently reported the presence of medical comorbidities (59.7% vs 46.9%) in comparison to men from the general population (Table 1 and Supplementary Table 4). Further sociodemographic characteristics were broadly comparable between the groups.

**Table 1:** Participant characteristics for patients with metastatic prostate cancer and men from the general population included in the study

	Prostate cancer	General population
	(*n* = 77)	(*n* = 311)
Median age, yr (IQR) {range}	73 (65–77) {51–86}	64 (56.5–71) {45–80}
Age group, *n* (%)		
45–64 yr	18 (23.4)	156 (50.2)
≥65 yr	59 (76.6)	155 (49.8)
Median VAS score for current health status (IQR) {range}	75 (65–86) {33–100}	85 (80–90) {15–100}
Data missing, *n* (%)	0 (0)	49 (15.8)
Comorbidity burden, *n* (%)		
At least one comorbidity	46 (59.7)	146 (46.9)
Hypertension a	24 (52.2)	96 (65.8)
Diabetes mellitus a	14 (30.4)	28 (19.2)
Cardiovascular disease a	20 (43.5)	41 (28.1)
Chronic respiratory disease a	4 (8.7)	19 (13.0)
Chronic kidney disease a	4 (8.7)	6 (4.1)
Chronic liver disease a	0 (0.0)	3 (2.1)
Other cancer diagnosis a	3 (6.5)	4 (2.7)
Smoking status, *n* (%)		
Nonsmoker	72 (93.5)	253 (82.1)
Smoker	5 (6.5)	55 (17.9)
Data missing	0 (0)	3 (1.0)
Education, *n* (%)		
None or mandatory school	4 (5.3)	3 (1.0)
Vocational training or baccalaureate	38 (50.0)	166 (53.4)
Higher technical school or college	14 (18.4)	72 (23.2)
University degree or doctorate	20 (26.3)	70 (22.5)
Data missing	1 (1.3)	0 (0)
Employment status, *n* (%)		
Employed or self-employed	15 (19.7)	139 (44.7)
Retired	59 (77.6)	163 (52.4)
Permanently on sick leave or without work	2 (2.6)	9 (2.9)
Data missing	1 (1.3)	0 (0)
In a partnership, *n* (%)	72 (94.7)	255 (82.8)
Data missing	1 (1.3)	3 (1.0)
Widowed and/or divorced, *n* (%)	15 (20.8)	57 (18.6)
Data missing	5 (6.5)	5 (1.6)
Has dependents, *n* (%)	6 (7.9)	62 (20.0)
Data missing	1 (1.3)	1 (0.3)
Place of residence, *n* (%)		
In the city	12 (15.6)	69 (22.2)
In a suburb	25 (32.5)	100 (32.2)
In the countryside	40 (51.9)	142 (45.7)
Language region, *n* (%)		
German-speaking	52 (67.5)	159 (51.1)
French-speaking	24 (31.2)	103 (33.1)
Italian-speaking	1 (1.3)	49 (15.8)
Nationality, *n* (%)		
Swiss	69 (89.6)	292 (93.9)
Non-Swiss	8 (10.4)	19 (6.1)
Current PC stage, *n* (%)		
Metastatic hormone-sensitive PC	57 (74.0)	–
Metastatic castration-resistant PC	20 (26.0)	–
Median time since diagnosis, yr (IQR) {range}	5 (2.2–10) {0–20}	–
Data missing, *n* (%)	3 (3.9)	
Median time since metastasis, yr (IQR) {range}	3 (2–6) {0–16}	–
Data missing, *n* (%)	5 (6.5)	–
Bone metastases present, *n* (%)	54 (70.1)	–
Currently receiving Tx, *n* (%)	74 (96.1)	–
Median time since starting current Tx, yr (IQR) {range}	2 (1–3) {0–13}	–
Data missing, *n* (%)	7 (9.1)	
Ever experienced adverse effects, *n* (%)	49 (69.0)	–
Data missing	6 (7.8)	
Experienced pain due to PC in the past 2 wk, *n* (%)	14 (18.7)	–
Data missing	2 (2.6)	
Any personal or professional experience with cancer, *n* (%)	–	236 (76.1)
Data missing	–	1 (0.3)

IQR = interquartile range; PC = prostate cancer; Tx = treatment; VAS = visual analog scale.

a Percentage among those reporting at least one comorbidity.

In the mPC group, 74.0% had mHSPC and 26.0% had mCRPC. 96.1% reported receipt of treatment for mPC, with a median time on current treatment of 2 yr (interquartile range [IQR] 1–3). While all reported having experience with treatment in the metastatic setting, 69.0% reported ever having experienced adverse effects from treatment. Among men from the general population, 76.1% stated that they have personal or professional experiences with cancer.

### 3.2 Assessment of internal validity

Almost all participants responded correctly to the dominance test (*n* = 374, 97.1%) and considered both alternatives in their responses (*n* = 7, 1.8% consistently chose either alternative; Supplementary Table 5). Twelve participants (3.1%) always chose the treatment with higher or equal survival benefit, while none based all choices on another attribute. The patients with mPC spent a median of 43 min (IQR 29–61) on the questionnaire, whereas men from the general population spent a median of 21 min (IQR 15–29). Overall, 80.0% (*n* = 311) took more than 15 min to complete the survey.

### 3.3 Participant preferences

**Fig. 2:**
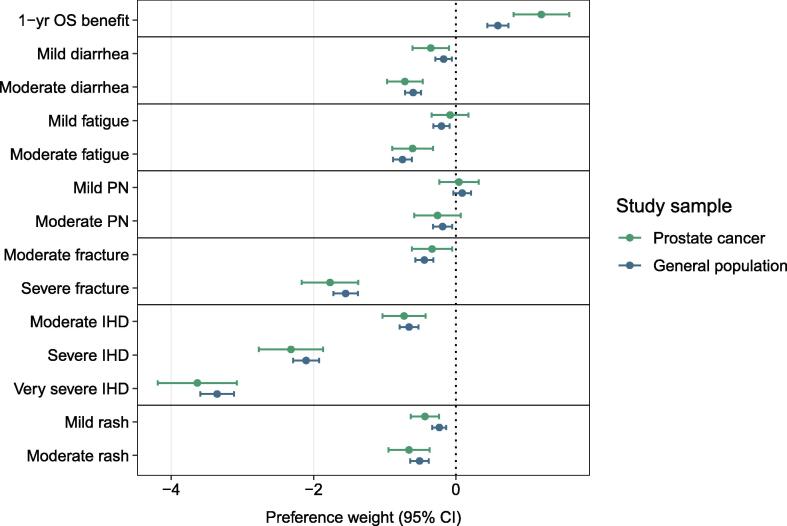
Preference weights regarding the benefits and harms of treatment for metastatic prostate cancer, taking into account preference heterogeneity between participants. Preference weights with 95% confidence intervals (CIs) were estimated for patients with metastatic prostate cancer and men from the general population using dummy-coded mixed logit models with overall survival (OS) as a random parameter. Preference weights represent the strength of preference relative to the reference level, with negative values representing a preference for averting the outcome. PN = peripheral neuropathy; IHD = ischemic heart disease.

There was very strong evidence that both patients with mPC (preference weight per additional year of survival 1.20, 95% confidence interval [CI] 0.81–1.59; *p* < 0.001) and men from the general population (preference weight 0.59, 95% CI 0.44–0.74; *p* < 0.001) had a preference for experiencing a survival benefit (Fig. 2 and Supplementary Table 6). Preference weights can be interpreted as the strength of preference for a benefit or a harm outcome relative to the respective reference level (ie, 4.5 yr of survival or the absence of the adverse effect), with negative values representing a preference for averting the outcome. Comparison of the participant samples revealed strong evidence that patients with mPC had a stronger preference for survival in comparison to men from the general population (test for difference: *p* = 0.004).

With respect to harm outcomes, there was strong evidence that participants from both populations had a preference for averting diarrhea, fractures, ischemic heart disease, and rash at all severity levels, as well as moderate fatigue (Fig. 2 and Supplementary Table 6). For mild fatigue and mild and moderate peripheral neuropathy, evidence of a preference for averting the outcome was insufficient among patients with mPC. Overall, there was no evidence of a difference in preferences between participant samples for any of the harm outcomes.

The number of months of survival that participants were willing to trade against averting different adverse effects differed between the mPC and general population groups (Table 2). Among patients with mPC, willingness to trade ranged from 1 mo for mild fatigue to 36 mo for very severe ischemic heart disease. The range was from 3 mo for mild diarrhea to 68 mo for very severe ischemic heart disease among general population participants.

**Table 2:** Maximum acceptable survival time in months that participants would be willing to trade against foregoing each of the possible harms of treatment for metastatic prostate cancer at different levels of severity: Marginal rates of substitution were estimated using mixed logit models with overall survival as a random parameter

Treatment-related effect	Prostate cancer		General population	
	(*n* = 77)		(*n* = 311)	
	MRS, mo (95% CI)	*p* value	MRS, mo (95% CI)	*p* value
Diarrhea				
Mild	4 (1–6)	0.012	3 (1–6)	0.007
Moderate	7 (4–10)	<0.001	12 (9–16)	<0.001
Fatigue				
Mild	1 (−2 to 3)	0.54	4 (2–7)	<0.001
Moderate	6 (3–9)	<0.001	15 (11–19)	<0.001
Peripheral neuropathy				
Mild	0 (−3 to 2)	0.76	−2 (−4 to 1)	0.18
Moderate	3 (−1 to 6)	0.11	4 (1–7)	0.006
Fracture				
Moderate	3 (1–6)	0.016	9 (6–12)	<0.001
Severe	18 (12–23)	<0.001	31 (24–39)	<0.001
Ischemic heart disease				
Moderate	7 (4–11)	<0.001	13 (9–17)	<0.001
Severe	23 (16–30)	<0.001	43 (32–53)	<0.001
Very severe	36 (26–47)	<0.001	68 (52–85)	<0.001
Rash				
Mild	4 (2–7)	<0.001	5 (2–7)	<0.001
Moderate	7 (3–10)	<0.001	10 (7–14)	<0.001

CI = confidence interval; MRS = marginal rate of substitution.

Evaluation of preference heterogeneity across participants revealed strong evidence of relevant variability in participants’ survival-related preferences both among patients with mPC (SD for preference weight 1.31, 95% CI 0.94–1.68; *p* < 0.001) and among men from the general population (SD for preference weight 1.04, 95% CI 0.89–1.19; *p* < 0.001; Supplementary Table 6). While preference heterogeneity was higher among patients with mPC in absolute terms, evidence of a difference between samples was insufficient (test for difference: *p* = 0.19). Overall, the findings were consistent throughout different sensitivity analyses excluding individuals who failed internal validity assessments, using different models for analysis, and using a propensity score–matched participant subsample for comparisons between population samples (Supplementary Tables 7–12).

### 3.4 Subgroup analyses

**Fig. 3:**
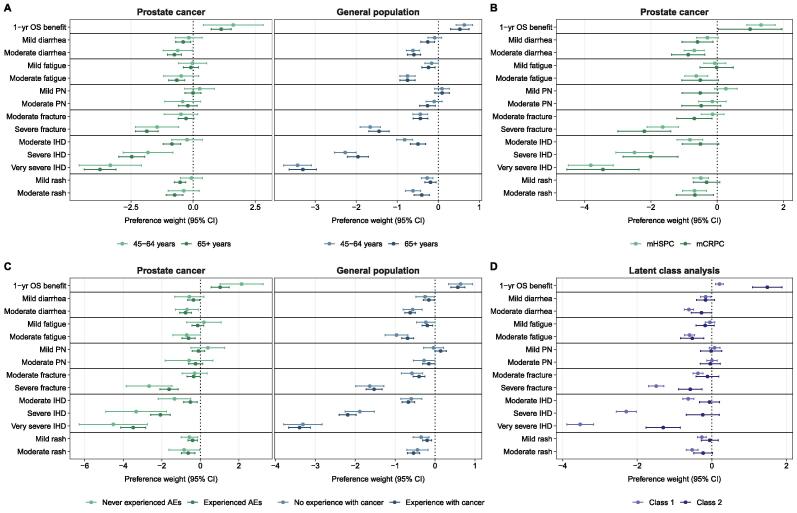
Preference weights regarding the benefits and harms of treatment for metastatic prostate cancer (mPC) from subgroup and experimental latent class analyses, taking into account preference heterogeneity between participants. Preference weights with 95% confidence intervals (CIs) were estimated separately for the respective subgroups using dummy-coded mixed logit models with overall survival (OS) as a random parameter. Preference weights represent the strength of preference relative to the reference level, with negative values representing a preference for averting the outcome. (A) Preferences in both samples stratified by age group. (B) Preferences among patients with mPC stratified by disease stage. (C) Preferences among patients with mPC and men from the general population stratified by whether they had prior or current experiences with treatment adverse effects (AEs) or personal or professional experiences with cancer, respectively. (D) Preferences in the two groups identified in latent class analysis, demonstrating a preference either for averting AEs while foregoing potential survival benefits (class 1), or for longer survival while accepting AEs (class 2). PN = peripheral neuropathy; IHD = ischemic heart disease.

In subgroup analyses stratified by age group, there was insufficient evidence of a difference in survival-related preferences between men aged 45–64 yr and men aged ≥65 yr, both in the mPC cohort (preference weight 1.61 [95% CI 0.41–2.81] vs 1.13 [95% CI 0.73–1.53]; test for difference: *p* = 0.45) and in the general population cohort (preference weight 0.63 [95% CI 0.43–0.83] vs 0.53 [95% CI 0.30–0.75]; test for difference: *p* = 0.48; Fig. 3 and Supplementary Tables 13 and 14). There was insufficient evidence of a difference in survival-related preferences between patients with mHSPC and those with mCRPC (preference weight 1.32 [95% CI 0.90–1.75] vs 0.99 [95% CI 0.03–1.96]; test for difference: *p* = 0.54). While there was a relevant difference in absolute preference weights in the mPC cohort between patients with and without prior experiences of adverse effects, statistical evidence of a difference was insufficient (preference weight 1.03 [95% CI 0.57–1.49] vs 2.14 [95% CI 1.03–3.25]; test for difference: *p* = 0.07). Finally, there was no evidence of a difference in survival-related preferences in the general population cohort between men with and without personal or professional experiences with cancer (preference weight 0.57 [95% CI 0.39 to 0.74) vs 0.64 [95% CI 0.34 to 0.94]; test for difference: *p* = 0.69).

### 3.5 Latent class analysis

The latent class analysis identified two groups with different sets of preferences among study participants, with strong evidence of a difference between groups (test for difference in survival-related preferences between classes: *p* < 0.001; Fig. 3 and Supplementary Table 15). The first group, including 76.0% of participants (class 1, *n* = 295), appeared to have a strong general preference for averting adverse effects and a lower preference for improvement in survival (while there was still strong evidence of a preference for survival benefits). The smaller second group (class 2, 24.0%, *n* = 93) had a strong preference for survival and showed a lower preference for averting adverse effects of treatment. Analysis of the distribution of participant characteristics in the two groups revealed no evidence that specific characteristics were associated with membership of either group according to descriptive and multivariable logistic regression analyses (Table 3).

**Table 3:** Participant characteristics in the two groups with different preference sets identified via latent class analyses and association of participant characteristics with membership of the group strongly preferring survival (class 2) a

	Participants, *n* (%)		Association with	
	Class 1: averting AEs	Class 2: survival	membership of class 2	
	(*n* = 295)	(*n* = 93)	OR (95% CI)	*p* value
Men from the GP	241 (81.7)	70 (75.3)	Reference	
Patients with metastatic PC	54 (18.3)	23 (24.7)	1.39 (0.73–2.61)	0.314
Mean age, yr [SD] {range}	64.9 [9.6] (45–86)	65.3 [9.2] (46–85)		
Median age, yr (IQR)	66.0 (58.0–72.5)	67.0 (58.0–73.0)	1.00 (0.97–1.03) b	0.828
Age group				
45–64 yr	135 (45.8%)	39 (41.9%)	Reference	
≥65 yr	160 (54.2%)	54 (58.1%)	1.09 (0.64–1.87)	0.756
Mean VAS score [SD] {range}	82.7 [12.8] (15–100)	80.8 [13.5] (33–100)		
Median VAS score (IQR)	85.0 (75.0–90.0)	82.0 (75.0–90.0)	0.99 (0.97–1.01) b	0.467
Data missing	38 (12.9)	11 (11.8)		
At least one comorbidity present	144 (48.8)	48 (51.6)	1.01 (0.59–1.71)	0.977
Hypertension	87 (29.5)	33 (35.5)		
Diabetes mellitus	27 (9.2)	15 (16.1)		
Cardiovascular disease	47 (15.9)	14 (15.1)		
Chronic respiratory disease	18 (6.1)	5 (5.4)		
Chronic kidney disease	8 (2.7)	2 (2.2)		
Chronic liver disease	3 (1.0)	0 (0.0)		
Other cancer diagnosis	4 (1.4)	3 (3.2)		
Smoking status				
Nonsmoker	248 (84.6)	77 (83.7)	Reference	
Smoker	45 (15.4)	15 (16.3)	0.81 (0.36–1.66)	0.583
Data missing	2 (0.7)	1 (1.1)		
Education				
None or mandatory school	3 (1.0)	4 (4.3)	Reference	
Vocational training or baccalaureate	154 (52.4)	50 (53.8)	0.19 (0.02–1.06)	0.067
Higher technical school or college	65 (22.1)	21 (22.6)	0.17 (0.02–0.99)	0.057
University degree or doctorate	72 (24.5)	18 (19.4)	0.13 (0.02–0.77)	0.030
Data missing	1 (0.3)	0 (0)		
Employment status				
Employed or self-employed	119 (40.5)	35 (37.6)	Reference	
Retired	166 (56.5)	56 (60.2)	1.50 (0.68–3.42)	0.318
Permanent sick leave or without work	9 (3.1)	2 (2.2)	0.80 (0.11–3.52)	0.785
Data missing	1 (0.3)	0 (0)		
In a partnership	248 (84.9)	79 (85.9)	0.87 (0.44–1.80)	0.689
Data missing	3 (1.0)	1 (1.1)		
Divorced and/or widowed	239 (82.4%)	67 (77.0%)	1.55 (0.82–2.84)	0.164
Data missing	7 (2.4%)	3 (3.2%)		
Has dependents	47 (16.0%)	21 (22.6%)	1.60 (0.79–3.15)	0.176
Data missing	2 (0.7)	0 (0)		
Place of residence				
In the city	60 (20.3)	21 (22.6)	Reference	
In a suburb	97 (32.9)	28 (30.1)	1.03 (0.50–2.20)	0.930
In the countryside	138 (46.8)	44 (47.3)	1.11 (0.57–2.25)	0.757
Language region				
German-speaking	158 (53.6)	53 (57.0)	Reference	
French-speaking	98 (33.2)	29 (31.2)	0.91 (0.54–1.53)	0.734
Italian-speaking	39 (13.2)	11 (11.8)	NE	–
Nationality				
Swiss	274 (92.9)	87 (93.5)	Reference	
Non-Swiss	21 (7.1)	6 (6.5)	0.89 (0.28–2.40)	0.828
**PC-specific characteristics**^c^	(*n* = 54)	(*n* = 23)		
Current PC stage				
Metastatic hormone-sensitive PC	40 (74.1)	17 (73.9)	Reference	
Metastatic castration-resistant PC	14 (25.9%)	6 (26.1%)	0.94 (0.27–2.98)	0.922
Mean time since Dx, yr [SD] (range)	6.8 [5.4] (0–20)	6.5 [5.4] (1–19)		
Median time since Dx, yr (IQR)	5.0 (3.0–10.0)	5.0 (2.0–9.0)	1.00 (0.90–1.11) b	0.984
Data missing	1 (1.9)	2 (8.7)		
Mean time since Mx, yr [SD] (range)	4.5 [3.9] (0–16)	4.2 [3.1] (1–11)		
Median time since Mx, yr (IQR)	3.0 (2.0–6.0)	3.5 (2.0–6.0)	0.99 (0.84–1.14) b	0.855
Data missing	2 (3.7)	3 (13.0)		
Bone metastases present	40 (74.1)	14 (60.9)	0.53 (0.18–1.57)	0.247
Currently receiving Tx	52 (96.3)	22 (95.7)	0.60 (0.05–13.88)	0.694
Ever experienced AEs	34 (68.0)	15 (71.4)	0.89 (0.27–3.09)	0.854
Experienced pain due to PC in past 2 wk	8 (15.1)	6 (27.3)	2.11 (0.57–7.68)	0.251
**GP-specific characteristics**^d^	(*n* = 241)	(*n* = 70)		
Any PPE with cancer	187 (77.9)	49 (70.0)	0.64 (0.34–1.24)	0.176
Data missing	1 (0.4%)	0 (0%)		

AEs = adverse effects; CI = confidence interval; Dx = diagnosis; GP = general population; IQR = interquartile range; Mx = metastasis; NE = not estimable; PC = prostate cancer; PPE = personal or professional experience; OR = odds ratio; SD = standard deviation; Tx = treatment; VAS = visual analog scale for current health status.

a Class 1 had a preference for averting adverse effects, while class 2 had a strong preference for a survival benefit. Association analyses are based on multivariable logistic regression models adjusted for sample (except for within-sample associations), age (except for age group), current health status, and the presence of at least one comorbidity.

b Per unit increment.

c Analysis restricted to patients with metastatic prostate cancer (*n* = 77).

d Analysis restricted to men from the general population (*n* = 311).

## 4 Discussion

### 4.1 Main findings

In this DCE preference study of patient and general population preferences regarding mHSPC treatment, we found that outcome preferences between patients suffering from mPC and men at risk of developing prostate cancer relevantly differ, with a stronger preference for survival benefits among patients with mPC overall. Furthermore, we found substantial heterogeneity in preferences between individuals, and identified two distinct groups of individuals strongly preferring either longer survival or the absence of adverse effects. Meanwhile, we did not find specific participant characteristics associated with belonging to either group, or evidence of differences between subgroups for age, disease stage, experiences with adverse effects, or personal or professional experiences with cancer.

Our findings suggest that patient preferences may have a relevant impact on treatment choices in mHSPC. The study shows that the potential adverse effects and their impact on HRQoL need to be equally considered as potential survival benefits. Indeed, the group of participants with a stronger preference for the absence of adverse effects was substantially larger than the group strongly preferring survival. While this does not mean that survival is not important for all patients (there was strong evidence of a survival preference in both groups), it indicates that the balance of benefits and harms is relevant for patients and that decision-making solely based on survival outcomes from clinical trials is likely to be inappropriate. It is thus critical that trials collect and fully report all relevant data, especially regarding adverse effects and HRQoL. Further investigations are necessary to determine the preference sensitivity of decisions in this context, combining information on patient preferences, real-world risks, and treatment effects related to the benefits and harms of mHSPC treatment. Our findings also suggest that preferences are difficult or even impossible to predict for an individual patient. Hence, our study provides strong evidence on the importance of considering and discussing individual patient preferences when making decisions regarding mPC treatment in clinical practice.

### 4.2 Findings in context

Previous studies have quantitatively [22], [23], [24], [25], [26], [27], [28], [41], [42] and qualitatively [29], [30], [43], [44], [45], [46], [47] investigated patient preferences related to mPC treatment. While these studies evaluated a wide range of different potential benefits, harms, and other aspects of treatment, evidence regarding the most important benefits and harms of treatment remains unclear [20], [21]. Previous studies primarily focused on identifying attributes of the highest importance to patients, which may help to guide treatment discussions in clinical practice [21], [22], [23], [24], [25], [26], [27], [41]. However, discussions about preferences between patients and physicians are most likely to improve patient-centered decision-making in contexts in which there is relevant preference heterogeneity (ie, potentially preference-sensitive decisions) [2], [21], [32]. In contrast to previous studies, we investigated and demonstrated the presence of preference heterogeneity in the context of mHSPC, thereby providing evidence that no single attribute is likely to be pivotal for treatment decisions in this context.

Whether the results from our study can be generalized to other disease contexts remains unclear. One previous study investigating patient preferences related to prostate cancer screening found substantial preference heterogeneity between participants [48]. Given the consistency of our results across participant samples and mPC disease stages, it may be reasonable to assume that substantial preference heterogeneity also exists more generally in mPC. Depending on their individual preferences combined with personal circumstances, life expectancy, and disease characteristics, some men with mHSPC may prefer to forego systemic treatment to avoid its potential harms. Therefore, while further research across different stages in mPC is necessary, guidelines in this context should ensure that they are sensitive and adaptive to differences in preferences between patients.

At a methodological level, preference heterogeneity is frequently discussed and statistical models accounting for such heterogeneity are often applied in studies [49], [50]. However, preference heterogeneity is rarely directly addressed or reported quantitatively in the literature [49], [50]. Furthermore, discussions are ongoing about whether it is most appropriate to measure the preferences of individuals from the general population at risk of facing the decision later, patients currently facing the decision, or patients with past experiences with the decision and its consequences [2], [31]. We attempted to address these questions by explicitly evaluating heterogeneity and comparing preferences between men at risk and men with past experiences, demonstrating relevant differences between individuals and populations. On the basis of our findings, future preference studies may benefit from a more comprehensive evaluation of preference heterogeneity.

Given the increasing interest in preference research to guide clinical decisions, regulatory assessment, and industry processes [2], [6], [7], [31], [49], it is important to consider how preference information from studies is used to guide clinical or policy decisions. Study designs and methods may differ according to their specific objectives and need to be interpreted in light of the respective stage along the medical product life cycle and the processes that should be informed [2], [21], [51], [52]. This study was designed to inform clinical decisions and to gather experiences for the use of preference information in benefit-harm assessment and health technology assessment, similar to case studies by the Innovative Medicines Initiative Patient Preferences in Benefit Risk Assessments During the Drug Life Cycle (IMI-PREFER) consortium [7]. While further experiences and methodological developments are necessary, findings from this study strongly support the implementation of shared decision-making based on patient preferences and may serve as a basis for developing clinical decision-making tools for clinical practice.

### 4.3 Limitations

Some limitations need to be considered when interpreting the results from this study. First, the recruitment strategies we used may have led to selection effects. While this may have influenced our results, the direction of potential biases is difficult to estimate. Since we could not collect data for individuals not participating in this study, we were unable to evaluate potential differences between participants and men not participating in the study. Furthermore, we sought the preferences of men residing in Switzerland only, whose preferences may be culturally different to those of men in other countries or of other ethnicities [53], [54], [55]. We did not ask for information on ethnicity or specific cultural elements beyond the language region in our study. In addition, patient preferences may vary across other health care contexts with differing access to care or in populations with different levels of baseline comorbidity. However, average preference weights corresponded approximately to what we had expected on the basis of previous studies conducted in other countries. Moreover, we deem it unlikely that a more representative or a more international sample of patients with mPC or the general population would have relevantly altered our findings regarding the presence of preference heterogeneity. Hence, we also consider our key results to be broadly generalizable in an international context. Second, we did not reach the desired size for the mPC sample (discussed in the Supplementary material). As a result, statistical power may have been too low to detect differences in preference heterogeneity in comparison to the general population or in preferences between patient subgroups with different disease stages or adverse effect experiences. Third, it is possible that the cognitive burden of the DCE may have led to inconsistent choices, declining concentration, or nonparticipation by individuals who are older or cognitively impaired. While we aimed to enroll as broad a study population as possible and ensure adequate preparation, instruction, support, and time for completion of the questionnaires, this may still have affected our results. Finally, as is common in DCEs, stated choices in hypothetical scenarios may not reflect the true choices of participants, and it is possible that other attributes not included in the DCE (ie, further benefit and harm outcomes, or other aspects such as mode of administration or cost of treatment) may also have a relevant impact on patients’ treatment decisions.

## 5 Conclusions

This study demonstrated relevant differences in preferences between individuals regarding the attributed benefits and harm of treatment for mHSPC. This information is crucial for clinical practice and the development of clinical practice guidelines, since it highlights the importance of explicitly taking patients’ individual preferences into account when making patient-centered treatment decisions. The study adds evidence on preference heterogeneity in the context of mPC, which may be important for the approval of novel treatments and health technology assessment. Future research may draw from this work to develop clinical decision-support tools and examine preference heterogeneity in other cancer settings.

  ***Author contributions***: Dominik Menges had full access to all the data in the study and takes responsibility for the integrity of the data and the accuracy of the data analysis.

  *Study concept and design*: All authors.

*Acquisition of data*: Menges, Piatti.

*Analysis and interpretation of data*: All authors.

*Drafting of the manuscript*: Menges.

*Critical revision of the manuscript for important intellectual content*: All authors.

*Statistical analysis*: Menges, Braun.

*Obtaining funding*: Menges, Puhan.

*Administrative, technical, or material support*: Menges, Piatti.

*Supervision*: Puhan.

*Other* (*participant recruitment*): Menges, Piatti, Omlin, Cathomas, Benamran, Fischer, Iselin, Küng, Lorch, Prause, Rothermundt, O’Meara Stern, Zihler.

*Other* (*project coordination*): Menges.

  ***Financial disclosures:*** Dominik Menges certifies that all conflicts of interest, including specific financial interests and relationships and affiliations relevant to the subject matter or materials discussed in the manuscript (eg, employment/affiliation, grants or funding, consultancies, honoraria, stock ownership or options, expert testimony, royalties, or patents filed, received, or pending), are the following: Aurelius Omlin has received institutional fees for an advisory role for Astellas, AstraZeneca, Bayer, Janssen, Molecular Partners, MSD, Pfizer, Roche, and Sanofi Aventis, personal fees for an advisory role for Astellas, AstraZeneca, Bayer, Janssen, Merck, MSD, and Novartis, institutional research support from Teva and Janssen, and travel support from Astellas, Bayer, Janssen, and Sanofi Aventis, and participates in speaker bureaus for Astellas, Bayer, and Janssen. Richard Cathomas has acted in an advisory role for Astellas, AstraZeneca, Bayer, BMS, Debiopharm, Ipsen, Merck, MSD, Janssen, Pfizer, Roche, and Sanofi, and has received honoraria from Astellas, BMS, and Janssen. Stefanie Fischer has received institutional fees for an advisory role for Ipsen and for speaker bureau participation for Janssen. Christian Rothermundt has received institutional fees for a consulting/advisory role for Bayer (Schweiz), Bristol-Myers Squibb, Ipsen, MSD Oncology, and Pfizer, personal fees for a consulting/advisory role for Merck (Schweiz), travel expenses from PharmaMar, and institutional research funding from Astellas. Lukas Prause has a compensated advisory role for Bayer and has received travel support from Astellas. Alix O’Meara Stern has received institutional fees for an advisory role for Takeda, AbbVie, Novartis, and Bayer, and participates in a speaker bureau for Incyte. Max Lippuner is a patient representative and acting president of Europa Uomo Switzerland, which received project-connected financial support from AbbVie, Amgen (Schweiz), Astellas, AstraZeneca, Bayer (Schweiz), Debiopharm, Janssen-Cilag, MediService, MSD International, Myriad Genetics, Sanofi-Aventis (Schweiz), and Sanofi Genzyme. The remaining authors have nothing to disclose.

  ***Funding/Support and role of the sponsor*:** This study was funded by the Swiss Cancer Research Foundation (Stiftung Krebsforschung Schweiz; grant no. HSR-4950-11-2019) and the Swiss Cancer Foundation. The salary of Dominik Menges was covered by a fellowship from the Béatrice Ederer-Weber Foundation. The funding bodies had no role in the design and conduct of the study; collection, management, analysis, or interpretation of the data; the decision to publish; or preparation, review, or approval of the manuscript.

  ***Data sharing statement***: The data sets generated and analyzed during the project are available from the corresponding author on reasonable request.

  ***Acknowledgments***: We thank the LINK Institute for their professional support in realizing this study and in recruiting study participants from the general population. We also thank Europa Uomo Switzerland, the Swiss Cancer League (Krebsliga Schweiz), and EUPATI Switzerland for their support in establishing important links to ensure patient involvement and feedback in this research project. In addition, we thank Dr. Silke Gillessen and Dr. Ursula M. Vogl for their expert input on the study design and implementation of the main survey. Furthermore, we thank Karin Haefeli and Gabriela Manetsch-Dalla Torre for their administrative support at the recruiting study centers, and Sonja Rüegg for her administrative support at the coordinating study center. Finally, we thank all the study participants for their valuable time and support of this research project.
