# Supplementary material for: Patient and General Population Preferences Regarding the Benefits and Harms of Treatment for Metastatic Prostate Cancer: A Discrete Choice Experiment
Source: Eur Urol Open Sci. 2023 Mar 22;51:26–38. doi: 10.1016/j.euros.2023.03.001 (PMC10175729; doi:10.1016/j.euros.2023.03.001)
Supplement: Supplementary data 1 [file mmc1.pdf]

Supplementary Material

Patient and General Population Preferences Regarding the Benefits and Harms of Metastatic Prostate Cancer Treatment: A Discrete Choice Experiment

Dominik Menges[a*], Michela C. Piatti[a], Aurelius Omlin[b,c], Richard Cathomas[d], Daniel Benamran[e], Stefanie Fischer[b], Christophe Iselin[e], Marc Küng[f], Anja Lorch[g], Lukas Prause[h], Christian Rothermundt[b], Alix O'Meara Stern[i], Deborah Zihler[j], Max Lippuner[k], Julia Braun[a], Thomas Cerny[l,m], Milo A. Puhan[a]

[a] *Epidemiology, Biostatistics and Prevention Institute (EBPI), University of Zurich (UZH), Zurich, Switzerland*

[b] *Department of Medical Oncology and Hematology, Kantonsspital St. Gallen (KSSG), St. Gallen, Switzerland*

[c] *Onkozentrum Zürich, Zurich, Switzerland*

[d] *Division of Oncology/Hematology, Kantonsspital Graubünden (KSGR), Chur, Switzerland*

[e] *Department of Urology, Hôpitaux Universitaires Genève (HUG), Geneva, Switzerland*

[f] *Department of Oncology, Hôpital Cantonal Fribourg (HFR), Fribourg, Switzerland*

[g] *Department of Medical Oncology and Hematology, University Hospital Zurich (USZ), Zurich, Switzerland*

[h] *Department of Urology, Kantonsspital Aarau (KSA), Aarau, Switzerland*

[i] *Department of Oncology, Réseau Hospitalier Neuchâtelois (RHNe), Neuchâtel, Switzerland*

[j] *Department of Oncology, Hematology and Transfusion Medicine, Kantonsspital Aarau (KSA), Aarau, Switzerland*

[k] *Europa Uomo Switzerland, Ehrendingen, Switzerland*

[l] *Foundation Board, Cancer Research Switzerland (Krebsforschung Schweiz KFS), Bern, Switzerland*

[m] *Human Medicines Expert Committee (HMEC), Swissmedic, Bern, Switzerland*

* Corresponding author: Dominik Menges, MD PhD MPH (dominik.menges@uzh.ch)

Attribute and Attribute Level Selection

Rationale

This study aimed to evaluate the preferences of metastatic prostate cancer (mPC) patients and men from the general population with respect to the attributed benefits and harms of treatment for metastatic hormone-sensitive prostate cancer (mHSPC). It was designed to address both clinical and methodological questions and to ultimately inform a quantitative benefit-harm assessment to support patient-centered decision-making in clinical practice.

Clinical questions to be addressed were:

- What are the considerations and trade-offs that patients make regarding the expected benefits and potential harms of treatment when making treatment decisions for mHSPC with their treating physician?
- Is there relevant heterogeneity in preferences among affected men that may determine treatment choices?
- Are there relevant patient subgroups that differ in terms of their preferences?

Methodological questions to be addressed were:

- Are there differences in preferences when elicited from individuals that never faced the treatment decision themselves (men at risk of developing mPC) and affected individuals that have personal experiences with decision-making (men with mPC)?
- How can patient preferences best be elicited to inform quantitative benefit-harm assessment as a basis for the development of clinical guidelines and health technology assessment?
- What are successful models to quantitatively elicit patient preferences in Switzerland and what experiences may be valuable for future studies?

In line with the overarching aims of the study, we conducted a discrete choice experiment (DCE) to elicit preference weights and estimate the trade-offs involved in clinical decision-making. Various methods for eliciting preferences are available, which need to be adapted to the specific context and research question [1–

4]. DCEs are one of the most common preference elicitation approaches, in which participants are asked to make choices within repeated hypothetical scenarios to allow the estimation of relative preference weights and trade-offs regarding different aspects of treatment [3,5–7]. We designed the preference study in line with guidance by the International Society For Pharmacoeconomics and Outcomes Research (ISPOR) [5–7]. As such, the project followed a multi-step approach to determine the study design as well as the attributes and attribute levels used in the DCE, consisting of a qualitative exploration stage, pilot testing, and the main preference survey.

Approach

We based the selection of attributes (i.e., patient-relevant benefit and harm outcomes of treatment for mHSPC) and attribute levels for the DCE preference study on information retrieved during the qualitative exploration stage. This included the following:

- Systematic literature review of patient preference studies related to mPC treatment
- Patient and expert interviews on the most important benefits and harms of mPC treatment
- Additional information sources
    - Systematic literature review of the evidence on treatments for mHSPC
    - Investigation of European Medicines Association (EMA) and swissmedic product labels for docetaxel, abiraterone acetate, enzalutamide and apalutamide
    - Literature screening on patient preferences in general and in the context of mPC.

Systematic Literature Review of Patient Preference Studies in Advanced Prostate Cancer

Findings from the systematic literature review of patient preference studies related to treatment of mPC are reported elsewhere [8].

Patient and Expert Interviews

Methods

We conducted semi-structured phone interviews between December 2020 and February 2021 with 13 mPC patients that had personal experiences with treatment choices in the context of mHSPC, as well as with 6 clinical experts (five medical oncologists and one urologist) actively involved in the care of mHSPC patients. mPC patients were recruited via the patient forum of the Swiss Cancer League and the patient organization Europa

Uomo Switzerland, in collaboration with the European Patients' Academy on Therapeutic Innovation Switzerland (EUPATI CH). Since this approach yielded only few participants, patients were additionally recruited via one involved clinical expert. Interviewed clinical experts from Switzerland were contacted directly by the lead researchers. Electronic consent was obtained from all patients and all clinical experts provided oral consent for participation in the interviews. All interviews were conducted in German language, and no financial compensation or other incentives were offered for participation. We enrolled interview participants until we arrived at the prespecified sample size [9] and reached theoretical saturation.

The interviews aimed at exploring treatment outcome-related aspects that are relevant for treatment decision-making, as well as at identifying the most patient-relevant benefits and the most important adverse effects of treatments for mHSPC at different levels of severity (i.e., mild, moderate, severe adverse effects). In addition, factors determining the importance of harms and information on the process of making treatment decisions in mHSPC were elicited. Expert interviews additionally included questions regarding the importance of shared decision-making in clinical practice. The interview questions were developed by two involved researchers based on the aims of the study, the findings of the systematic literature review of patient preference studies in mPC, input by involved clinical experts, and additional information sources outlined above. Questions were then iteratively refined based on the collected information, leading to minor revisions of the wording and addition of further questions. All interviews were audio-recorded and transcribed.

The collected information from patient and expert interviews was analyzed using a framework analysis approach [10], including familiarization with the data, development of the thematic framework, coding, charting, and interpretation of the data. We defined the initial thematic framework based on previous findings from the qualitative exploration stage, which was discussed and iteratively refined by three researchers until agreement on an initial set of codes was reached. We then applied iterative coding using MAXQDA software (VERBI Corp., Berlin Germany) to categorize interview responses to corresponding themes. Conceptually related codes were grouped into thematic categories, and the thematic framework was iteratively refined until the final set of themes (n=19) and codes (n=121) was reached. We then summarized the data using charting based on themes and codes and analyzed patterns and associations of different themes between participants.

Results

Of 13 interviewed mPC patients, 4 (31%) had mHSPC and 9 (69%) had metastatic castration-resistant prostate cancer (mCRPC). Eight participants had initially been diagnosed with metastatic disease and participants were at a median of 6 years (interquartile range (IQR) 3–12 years) since diagnosis (Supplementary Table 1). All patients reported to have experienced or to experience one or more adverse effect of mHSPC treatment. Most commonly reported adverse effects were hot flushes (n=13, 100%), fatigue (n=6, 46%), dizziness (n=4, 31%), diarrhea or nausea/vomiting (n=3, 23%), and myocardial infarction (n=3, 23%). Among the 6 interviewed clinical experts, all were actively involved in the care of mPC patients, with a median of 8 years (IQR 6–20 years) of clinical experience.

Supplementary Table 1: Participant characteristics of metastatic prostate cancer patients participating in the semi-structured phone interviews during the qualitative exploration stage of the study.

	**mPC patients** (N=13)
**Age (years)**	
Median (IQR)	70.0 (66.0 to 73.0)
Range	64 to 81
**Current diagnosis**	
mHSPC	4 (31%)
mCRPC	9 (69%)
**Stage at time of first diagnosis**	
Localized	8 (62%)
Metastasized	5 (39%)
**Time since first diagnosis (years)**	
Median (IQR)	6 (3 to 12)
Range	1 to 18
**Current work status**	
Partially retired	1 (8%)
Retired	12 (92%)
**Current therapy received**	
ADT + enzalutamide	5 (39%)
ADT + abiraterone acetate	4 (31%)
ADT + radium-223	1 (8%)
ADT only	1 (8%)
Lutetium-177 PSMA	1 (8%)
Orchiectomy	1 (8%)
**Prior therapies received**[a]	

ADT + docetaxel	4 (31%)
ADT + enzalutamide	1 (8%)
ADT + bicalutamide	3 (23%)
ADT only	4 (31%)
ADT + radiotherapy	6 (46%)
Others	3 (23%)
**Adverse effects experienced**[b]	
Hot flushes	13 (100%)
Fatigue	6 (46%)
Dizziness	4 (31%)
Diarrhea or nausea/vomiting	3 (23%)
Myocardial infarction	3 (23%)
Peripheral sensory neuropathy	2 (15%)
Alopecia	2 (15%)
Osteoporosis	2 (15%)
Gynecomastia	2 (15%)
Loss of taste	2 (15%)
Depression	1 (8%)
Febrile neutropenia	1 (8%)
Hearing loss	1 (8%)
Hypothyroidism	1 (8%)
Loss of appetite	1 (8%)
Loss of physical fitness	1 (8%)
Skin rash	1 (8%)
Weight loss	1 (8%)

Legend: ADT: androgen deprivation therapy, IQR: interquartile range, mCRPC: metastatic castration-resistant prostate cancer, mHSPC: metastatic hormone-sensitive prostate cancer, mPC: metastatic prostate cancer, PSMA: prostate-specific membrane antigen. [a] Some participants may have received multiple therapies. [b] Some participants may have experienced multiple adverse effects that they attributed to treatment.

We defined five main content areas through the interviews: the patients' role in decision-making, factors that are important for decision-making, patient-relevant benefits and harms of mHSPC treatment, factors that determine the importance of harms, and possible improvements for the decision-making process. Overall, shared decision-making was seen as highly important in the context of mPC by all interviewed clinical experts. Both patients and experts stated that patients may take different roles (i.e., active, collaborative, or passive) in decision-making depending on their decision-making preferences. All but one interviewed patient were taking a more active or collaborative role, while shifts in decision-making preferences from passive to more active roles were described.

Regarding factors that are relevant for decision-making, patients stated that the information provided by their treating physician was important and determined their patient-provider relationship. Furthermore, the prospects of treatment success (e.g., lowering of prostate specific antigen (PSA) levels, stopping the spreading of cancer, or radiographic improvement) were crucial in treatment discussions. And third, the balancing of benefits and adverse effects of treatment were considered important, to allow patients to maintain as high a quality of life as possible. Experts mentioned similar factors, with the addition of mode of administration, treatment duration, time requirements of different modes of treatment, contraindications due to comorbidities, and patients' preconceptions about certain treatments (e.g., chemotherapy) also playing a role for mHSPC treatment decisions.

Regarding patient-relevant benefits of mHSPC treatment, patients mentioned treatment success, overall survival, absence of adverse effects, and health-related quality of life (HRQoL) as important benefit outcomes. Definitions of treatment success varied and included lowering of prostate specific antigen (PSA) levels, stabilization of the disease, or radiographic improvement. The absence of pain was further stated as an important benefit by one patient. Similarly, experts evaluated the most important benefit to be overall survival, followed by HRQoL, absence of adverse effects, and more generally treatment success. One expert evaluated HRQoL and absence of adverse effects to be more important in discussions with elderly patients than overall survival.

Regarding the potential harms of mHSPC treatment, patients evaluated myocardial infarction to be the most severe due to its medical consequences, potential physical limitations, and threat to life (Supplementary Figure 1A). Furthermore, fatigue was reported to be one of the most important adverse effects, especially in patients that were still actively participating in work life. Outcomes leading to a substantial short-term (diarrhea, nausea/vomiting) or longer-term (fractures) restriction in daily activities were also considered highly limiting. Meanwhile, patients evaluated hot flushes, stomatitis, and febrile neutropenia to have only little impact on HRQoL, with possible countermeasures (e.g., for hot flushes) being reported as an alleviating factor. The importance attributed to peripheral neuropathy appeared to depend on how severely interviewed patients had

themselves been affected by the outcome. Differences emerged in the evaluation by clinical experts (Supplementary Figure 1B), who evaluated fatigue, peripheral neuropathy, and febrile neutropenia to be particularly limiting for patients. The risk of infection with febrile neutropenia was especially mentioned with regards to the effect of the coronavirus disease 2019 (COVID-19) pandemic on patients. Peripheral neuropathy was seen as important due to its long-lasting duration and the lack of available treatment options. In contrast, experts viewed gastrointestinal adverse effects (diarrhea, nausea/vomiting) and myocardial infarction as less important adverse effects in the context of mHSPC, and several considered the additional risk due to mHSPC treatment as minimal. Stomatitis was also reported to have little impact on patients' HRQoL, and fractures were considered less important mainly due to the availability of countermeasures (e.g., anti-osteoporotic treatments). Similarly to patients', experts stated that hot flushes were, while frequent and bothersome, often less limiting the HRQoL of patients, since most were only mildly affected or had learned to cope well with them.

Supplementary Figure 1: Metastatic prostate cancer patients' (panel **A**) and clinical experts' (panel **B**) evaluation of the importance of different potential adverse effects of treatment for metastatic hormone-sensitive prostate cancer, and factors that determine the importance of treatment adverse effects based on patient and expert interviews (panel **C**).

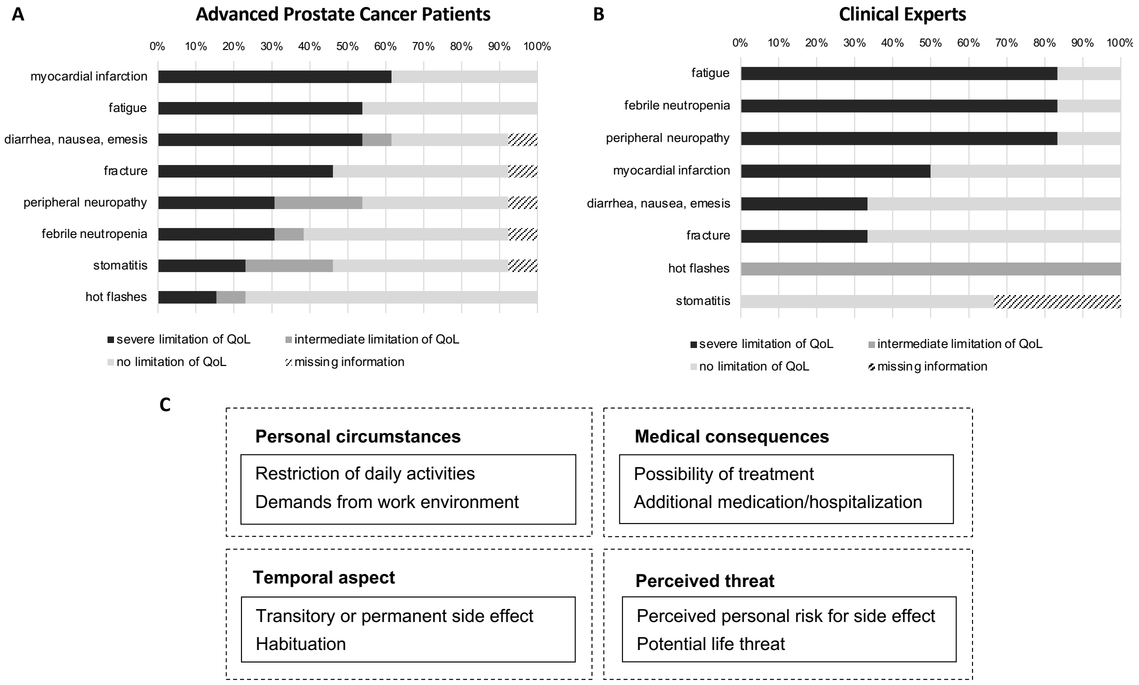

Factors that determine the importance of adverse effects were reported by patients to be potential limitations in daily activities, the possibility of taking countermeasures or availability of treatment, the perceived personal risk for the adverse effect, the adverse effects' duration (i.e., transitory (short-term) vs. permanent (long-term) effects), and its medical consequences (i.e., the need for additional medication, doctor visits, or hospitalization; Supplementary Figure 1C). Furthermore, potential habituation to certain adverse effects was mentioned as an additional factor. Experts mentioned broadly the same factors determining the importance of adverse effects, including limitations in daily activities, possibility of treatment, and perceived risk for experiencing an adverse effect. One expert stated that the combination of certain adverse effects may primarily determine the resulting limitations in HRQoL.

Discussion

In the patient and expert interviews, overall survival and treatment success more generally (without a specific definition) were most frequently mentioned as the most important benefits of mHSPC treatment. In terms of treatment harms, patients viewed fatigue, gastrointestinal adverse effects, myocardial and fractures as the most important, while hot flushes, stomatitis and febrile neutropenia were reported to be outcomes that had less impact on their decisions related to treatment. In contrast, experts more frequently reported fatigue, febrile neutropenia and peripheral neuropathy as important harms, while adverse effects with a lower additional risk due to treatment (e.g., myocardial infarction) and for which potential countermeasures or treatments are available (e.g., hot flushes, fractures) were considered less important. Overall, the interviews underscored that the expected benefits of treatment have to be weighted carefully against its potential harms when deciding on a treatment for mHSPC in clinical practice, in line with previous qualitative research conducted in this context [11].

Both patients and experts stated several factors that they considered as highly important when judging the importance of different adverse effects of mHSPC treatment, such as the severity of the adverse effects in terms of their limitation in daily life and their medical consequences, their duration and potential habituation, as well as available treatments or countermeasures. These findings stand in contrast to previous patient preference studies in the context of mPC. Within the systematic review conducted as part of this project [8], three out of seven identified quantitative preference elicitation studies included overall survival as a benefit outcome.

Furthermore, all but one of the identified six DCE studies defined harms at different levels of risk [12–16], and only one DCE study also used levels of severity in the definition of harm outcomes [17]. While we also found the perceived risk of a harm outcome as an important factor for decision-making, the severity and duration and the perception of the impact of potential adverse effects on patients' lives appeared to have a stronger impact based on the interviews. We considered this information as critical for the further design of the DCE conducted within this project.

Some limitations need to be considered when interpreting the results of the interview studies. First, we recruited participants through patient forums and organizations and an involved clinical expert. Patients that participated may have been more actively involved in their care and better informed about the benefits and harms of mHSPC treatment than the broader population of mPC patients. Furthermore, we recruited mCRPC patients and mHSPC patients with prior treatment experience. Hence, participating patients may have drawn from their personal experiences and perceptions when answering to the interview questions, which may have influenced their perception, especially regarding adverse effects (e.g., adverse effects that they may have experienced themselves). Last, we had initially planned to conduct in-person focus groups with a larger sample of participants. Due to the COVID-19 pandemic, we decided to conduct in-person interviews because of concerns about feasibility and potential infection risk. Meanwhile, the most important results concern the factors that determine the importance of adverse effects of treatment. Since we reached theoretical saturation in both patients and experts and both groups broadly agreed in the factors impacting decisions, we are convinced that we overall collected information that is representative of the general perceptions among men affected by mPC.

Additional Information Sources

Systematic Literature Review of Treatments for Metastatic Hormone-Sensitive Prostate Cancer

Findings from the systematic literature review and network meta-analysis of randomized controlled trials (RCTs) evaluating treatment with docetaxel, abiraterone acetate, enzalutamide or apalutamide in combination with androgen deprivation therapy in mHSPC are reported elsewhere [18].

Mapping

In a next step, we compiled and mapped the information retrieved in the qualitative exploration stage to create a framework for the selection of the final attributes and attribute levels for the discrete choice experiment. Important aspects considered were that they should be patient-relevant (i.e., either frequent or substantially impacting patients' daily lives), be distinctive between available treatments for mHSPC, and reflect different levels of severity and impact on patients' daily lives (i.e., medical consequences and duration). For benefit outcomes, we mapped candidate attributes (i.e., overall survival, HRQoL, pain control, progression-free survival, time to next treatment, time to symptomatic skeletal event, time to pain progression) according to their direct patient relevance (i.e., direct outcome versus surrogate outcome), as well as according to their potential overlap with harms of treatment (e.g., adverse effects impacting HRQoL).

For harm outcomes, we mapped candidate attributes (i.e., frequently occurring adverse effects or adverse effects severely impacting patients' lives; fatigue, hot flushes, rash, peripheral edema, hypertension, electrolyte disturbances, hepatic enzyme reactions or liver failure, nausea or vomiting, diarrhea, stomatitis, nail changes, alopecia, hypersensitivity, febrile neutropenia, peripheral sensory neuropathy, hypothyroidism, cognitive disorder, falls, fractures, cardiac arrhythmia, heart failure, ischemic heart disease, and seizures) according to their most frequent levels of severity based on data reported by the EMA and RCTs of docetaxel, abiraterone acetate, enzalutamide, and apalutamide in the context of mHSPC [18]. We determined to use levels of severity rather than risks since previous research has shown that risks are often poorly understood by study participants [19–21] and our interviews suggested that the severity of adverse effects is more important than its perceived risk. Furthermore, risk levels include an implicit assumption about the severity of an adverse effect [22], which may differ from individual to individual (e.g., due to past experiences or preconceptions) and thereby influence the results. We used the Common Terminology Criteria for Adverse Effects (CTCAE) classification as a basis for categorizing the severity of adverse effects [23], since it is well standardized and commonly applied in RCTs, and since severity grades are defined by their medical consequence and impact of the patients' lives. We evaluated both of these aspects to be desirable for later application of preference weights in quantitative benefit-harm assessment based on RCT data.

Selection and Definition

The selection and definition of the final attributes and attribute levels was guided by the ISPOR recommendations [5,6] and the following prespecified principles [9]:

- Attributes
    - Should be primarily focused on benefits and harms of treatment in order to inform benefit-harm assessment, not on other factors determining treatment preferences (e.g., mode of administration, costs, etc.)
    - Should be primarily focused on distinctive effects of docetaxel, abiraterone acetate, enzalutamide, and apalutamide (including required co-treatments, such as prednisone with abiraterone acetate), less on effects of concomitant androgen deprivation therapy
    - Should incorporate different factors influencing the importance of harm outcomes (as identified during the qualitative exploration stage; i.e., restriction in or interference with daily life, medical consequences, threat to life, possibility of treatment or countermeasures, duration, and habituation) explicitly in the outcome descriptions and should be balanced with respect to these factors
    - Should be balanced across the different adverse effect profiles of the available mHSPC treatments (i.e., docetaxel, abiraterone acetate, enzalutamide, and apalutamide)
    - Should not be strongly correlated or overlap
- Attribute levels
    - Should be easily understandable and interpretable for study participants
    - Should be based on evidence as far as possible and available, using realistic outcome descriptions, severity levels, and/or estimates in the context of mHSPC
    - Should reflect outcome preferences at different levels of severity, not preferences for outcome risks
    - Are selected based on an iterative process including expert feedback and patient feedback through pilot testing, and complemented with information from the systematic literature review of preference studies to enable comparability.

The final DCE included seven attributes consisting of one benefit and six harm outcomes with three to four attribute levels each. We defined the benefit attribute as overall survival benefit beyond the median survival time, in line with the approach of previous preference studies [14,17]. The rationale for using overall survival was that we considered it most directly patient-relevant (as opposed to surrogate outcomes such as progression-free survival), having little overlap with adverse effects (as opposed to HRQoL, with deadly adverse effects reported infrequently in RCTs [18]), and being most distinctive between treatments (as opposed to pain control). Based on data from published RCTs, we assumed 4 years to be the median survival time for patients newly diagnosed with mHSPC [18,24]. We then defined the attribute levels as a survival benefit of 6 months (total survival of 4.5 years), 1 year (5 years), and 2 years (6 years), which we deemed realistic treatment effects based on published RCT data [18,24].

Regarding treatment harms, we defined the adverse effects of diarrhea, fatigue, peripheral (sensory) neuropathy, fractures, ischemic heart disease, and rash as harm attributes used in the DCE. The rationale for selecting these outcomes over others were to cover the full spectrum of severity (i.e., mild to very severe adverse effects), to ensure a balance of adverse effects that are of importance with different mHSPC treatments (i.e., docetaxel, abiraterone acetate, enzalutamide, and apalutamide), and to ensure a balance of short-term and long-term adverse effects. We defined attribute levels in line with CTCAE grades, with mild adverse effects corresponding to grade 1, moderate to grade 2, severe to grade 3, and very severe to grade 4 adverse effects. We chose the severity levels that were most frequently reported in published RCTs for each of the harm attributes [18]. Diarrhea, fatigue, peripheral neuropathy and rash were defined with three levels (no, mild, and moderate adverse effects), fracture was defined with three levels reaching higher severity (no, moderate, and severe adverse effects), and ischemic heart disease was defined with four levels reaching highest severity (no, moderate, severe, and very severe adverse effects; Supplementary Table 2).

Supplementary Table 2: Treatment attributes and attribute levels included in the discrete choice experiment.

Benefit or harm outcome	Attribute	Attribute levels
Overall survival	Survival	4.5 years (4 years + additional 6 months)
		5 years (4 years + additional 1 year)
		6 years (4 years + additional 2 years)
Diarrhea	Diarrhea	No diarrhea
		Mild diarrhea
		Moderate diarrhea
Fatigue	Fatigue (feeling exhausted)	No fatigue
		Mild fatigue
		Moderate fatigue
Peripheral (sensory) neuropathy	Sensory disturbances	No sensory disturbances
		Mild sensory disturbances
		Moderate sensory disturbances
Fracture	Bone fracture	No fracture
		Moderate fracture
		Severe fracture
Ischemic heart disease	Cardiovascular disease	No cardiovascular disease
		Moderate cardiovascular disease
		Severe cardiovascular disease
		Very severe cardiovascular disease
Rash	Rash	No rash
		Mild rash
		Moderate rash

Detailed Description of Attributes and Attribute Levels

For each of the attributes and attribute levels, we developed a short outcome description. The descriptions included included aspects related to medical consequences, restrictions in daily life, possibility of treatment or countermeasures, and likely duration of the outcomes, corresponding to the selection principles outlined above (Supplementary Table 3). The outcome descriptions aimed to provide a common reference for all participants in order to reduce potential biases related to participants' preconceptions about the meaning of the attributes. They were developed by two involved researchers and iteratively refined based on the feedback of three further involved researchers, as well as of mPC patients and men from the general population during pilot testing.

All study participants were provided with the short outcome descriptions and asked to read them prior to completing the DCE. A short summary of the full outcome descriptions were provided within the displayed DCE choice task as a reminder on the key aspects (see example DCE choice task in the main article in Figure 1). Participants also had the possibility to revisit the full descriptions at all times during the DCE.

Supplementary Table 3: Outcome descriptions for all benefit and harm attributes and attribute levels (survival benefit or severity of adverse effects for harms) presented to study participants prior to and available during the discrete choice experiment. Descriptions were provided to participants in German, French, or Italian.

**Benefit**				
**Survival**<br><br>The average survival time with the disease is 4 years. This means that half of all individuals with the disease survive at least 4 years without additional treatment. However, the other half die before this time. The survival time varies greatly between affected persons and can therefore also be much longer or shorter. Please orientate yourself based on the average value of 4 years.	**4.5 years**<br><br>With the medication you live **6 months longer**.<br><br>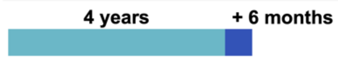	**5 years**<br><br>With the medication you live **1 year longer**.<br><br>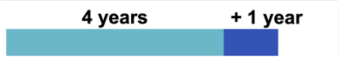	**6 years**<br><br>With the medication you live **2 years longer**.<br><br>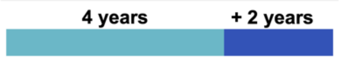	
**Side effects**				
**Diarrhea**<br><br>Diarrhea means that you have liquid or loose stools several times a day.	You have **no** diarrhea.	**Mild**<br><br>You have diarrhea for 1-2 days and need to go to the bathroom several times a day. However, you can leave the house.<br><br>Taking tablets brings an	**Moderate**<br><br>You have severe diarrhea for 3-4 days and need to go to the bathroom several times a day. You can hardly control the diarrhea and therefore cannot leave the house. Taking tablets brings only little improvement.	

		improvement.<br>After 2-3 days, you have normal bowel movements again and you have no lasting symptoms.	After 4-5 days, you have normal bowel movements again and you have no lasting symptoms.	
**Fatigue (feeling exhausted)**<br><br>Fatigue means that you suffer from severe tiredness, exhaustion or a general feeling of weakness.	You have **no** fatigue.	**Mild**<br><br>You often feel tired and sometimes have to lie down after midday.<br>The fatigue only slightly restricts you in your everyday life.<br><br><br>The symptoms can be slightly improved by exercise and sport, but there is no therapy for the symptoms.<br>The fatigue persists in the long term until the end of treatment.	**Moderate**<br><br>You feel tired all the time and can hardly get by without lying down after midday.<br>The fatigue strongly restricts you in your everyday life. You find little energy to get out of the house or meet acquaintances.<br>The symptoms can hardly be improved by exercise and sport and there is no therapy for the symptoms.<br>The fatigue persists in the long term until the end of treatment.	
**Sensory disturbance**<br><br>Sensory disturbances occur because of damage to the nerves. This can result in decreased sensation or numbness in the hands and feet, burning or tingling sensations, or an unsteadiness when standing.	You have **no** sensory disturbances.	**Mild**<br><br>You have mild discomfort in your hands and feet. You have a reduced feeling in your fingertips and find it more difficult than before to grasp small things.	**Moderate**<br><br>You have relevant discomfort in your hands and feet. Your fingers and feet feel numb. You have difficulty grasping things. From time to time you feel an bothersome burning sensation in both feet. You do not feel as steady on your	

		There is no therapy for the symptoms.<br>The sensory disturbances persist in the long term until the end of your life.	feet as you used to.<br>There is no therapy for the symptoms.<br>The sensory disturbances persist in the long term until the end of your life.	
**Bone fracture**<br><br>You can suffer a bone fracture either because of a weakened bone density (osteoporosis) or because of a fall. Certain medications can increase the risk of osteoporosis or falls.	You have **no** bone fracture.	**Moderate**<br><br>Due to a bruise, you break a rib. The fracture causes pain when breathing.<br>You are treated for this by your general practitioner with several painkillers.<br>For 3-4 weeks you are restricted in breathing and moving your upper body.<br>After that, you are completely well again.<br><br>In the long term, no discomfort remains. You need to take a medication to improve bone density.	**Severe**<br><br>Due to a fall, you break your thigh bone (femur). The fracture causes severe pain.<br>You undergo emergency surgery in hospital and are treated with strong painkillers.<br>You have to use crutches for 4 weeks and are not allowed to put any weight on your leg.<br>After that, you can slowly put weight on your leg again, but you are still restricted. You have to go to physiotherapy for several months. After 6 months, you are completely well again and can put your full weight on the leg.<br>In the long term, no discomfort remains. You need to take a medication to improve bone density.	
**Cardiovascular disease**	You have **no** cardiovascular disease.	**Moderate**	**Severe**	**Very severe**

Cardiovascular disease means that the heart vessels are narrowed due to calcification. This results in a poor blood flow to the heart and too little oxygen reaches the heart. Certain medications can increase the risk of cardiovascular disease. One treatment option is to dilate the heart vessels with a balloon and insert a small wire tube (stent) to improve the blood flow.		You feel a mild pressure on your chest during strenuous physical activity and you feel less able to perform.<br>You go to your general practitioner, who arranges for various tests to be carried out. A stress test on a bicycle is performed.<br><br>Your physician prescribes three additional medications, which you must take daily. In addition, you have to adjust your lifestyle.<br><br>You remain slightly limited in your physical performance. You have an increased risk of suffering a heart attack.	You suddenly feel a severe pain in your chest and feel unwell.<br><br>You go to the emergency at the hospital, where various examinations are carried out. A heart catheter examination is performed and a stent is inserted.<br><br>After a few days of monitoring in the hospital, you feel well again and can go home. You need to see your general practitioner several times for check-ups and need to restrict yourself for a few more weeks. You have to take four additional medications.<br><br>You remain slightly limited in your physical performance. You have an increased risk of suffering another heart attack.	You suddenly feel a severe pain in your chest, have difficulty breathing and feel on the verge of collapse.<br>You are taken to the hospital by ambulance, where various investigations are carried out. An emergency heart catheter examination is performed and two stents are inserted.<br>You are transferred to the intensive care unit for a few days and have to stay in hospital for another two weeks for monitoring. After that, you feel well again and can go home. You have to see your general practitioner several times for check-ups and take part in an outpatient rehabilitation programme for three months. You have to take four additional medications.<br>You remain significantly limited in your physical performance and you complain of swollen legs from time to time. You have an increased risk of suffering another heart attack.
**Rash**	You have **no** rash.	**Mild**	**Moderate**	

You react to the medication with redness or itching of the skin.		You have a mild rash that affects your belly. You experience mild itching in the reddened areas.<br><br>Your general practitioner prescribes an ointment which you apply to the reddened areas for 2 weeks.<br><br>This will make the rash disappear. In the long term, you have no lasting symptoms.	You have a relatively severe rash that affects the frontal part of your entire torso. You experience a bothersome itching sensation in the reddened areas.<br>Your general practitioner prescribes tablets, which you take regularly for 2 weeks and after that as needed. You are also given an ointment which you apply to the reddened areas for 4 weeks. The rash is cosmetically disturbing, so you leave your house less during this time.<br>Over time, the rash will slowly disappear. In the long term, you have no lasting symptoms.	

Pilot Testing

Experimental Design

The experimental design during pilot testing corresponded to the one used in the main preference survey, since no major adaptations to the design were undertaken based on pilot testing. We constructed the DCE using a Bayesian D-efficient design derived through a coordinate exchange algorithm implemented in the *idefix* (v1.0.3) package in *R* [25,26]. We applied predetermined priors to produce 50 candidate sets of 15 choice tasks, from which we selected the DCE design which ensured a high design efficiency and attribute balance. Assuming a normal distribution, we used the following priors for the algorithm, calculated as the log of the assumed odds of choosing either treatment based on each attribute level: log(1.33)=0.285 for 5 years of survival, log(2.0)=0.693 for 6 years of survival, log(0.8)=-0.223 for mild, log(0.6)=-0.511 for moderate, log(0.4)=-0.916 for severe, and log(0.2)=-1.609 for very severe adverse effects. Priors were of generic nature, since we deemed the estimates from previous preference studies not to be fully applicable to the design of our study due to important differences in the definition of attributes [8]. The (Bayesian) D-efficiency of the final design was 97.6%. Based on the results of the pilot testing, we considered our prior assumptions to be relatively correct, while relevant uncertainty remained due to the limited sample size. Furthermore, we assumed that preference heterogeneity may be present. Therefore, we chose not to update the priors for the main survey.

Participant Recruitment

For pilot testing, we recruited a purposive sample of 12 mPC patients among the sample of individuals identified for the patient interviews during the qualitative exploration stage. Participants from the mPC patient sample were surveyed between 30 July 2021 and 16 November 2021. Men from the general population were recruited through the respondent panel of the involved Swiss social research social and market research institute (LINK), with 20 participants surveyed from 20 to 21 September 2021.

Subsequent Adaptations

Based on participants' feedback and experiences from pilot testing, minor adaptations were made to the wording of some outcome descriptions, one question related to the evaluation of participants' experiences with the DCE ("I was confident about my choice for most of the comparisons"), as well as questions related to disease history (specified that participants only had to provide the key data). Additional feedback on the wording of specific questions was obtained from experienced researchers of the involved social and market research institute. Further minor wording changes were implemented during translation of the questionnaire, in order to better align the wording across questionnaires in different languages.

Main Preference Survey

Participant Recruitment and Survey Timeframes

Supplementary Figure 2 depicts the enrolment process for mPC patients and men from the general population into the study.

mPC patients were recruited by involved clinical experts between 26 November 2021 and 24 May 2022 and surveyed between 14 December 2021 and 08 May 2022. Patients were recruited in seven participating study centers in the German- and French-speaking parts of Switzerland (Cantonal Hospital Aarau, Cantonal Hospital Fribourg, University Hospital Geneva, Cantonal Hospital Graubünden, Hospital Network Neuchâtel, Cantonal Hospital St. Gallen, University Hospital Zurich).

Men from the general population were recruited and surveyed between 29 November 2021 and 07 December 2021 through the respondent panel of the involved Swiss social and market research institute (LINK). General population participants were invited based on a sample stratified by age group (45–64 years, ≥65 years) and language region (German-, French- and Italian-speaking parts of Switzerland; prespecified as a ratio of 150:100:50 for participants speaking the respective languages, which

approximately corresponds to the share of the respective population sizes in Switzerland while ensuring minimum sample sizes in each stratum).

Study Administration Considerations

The original plan of the project was to perform the preference survey with prostate cancer patients as in-person interviews. The rationale was to allow a more direct explanation of the DCE choice tasks tailored to the cognitive abilities of the participating patients, provide additional support if needed, and allow a more in-depth cognitive debriefing. Due to the COVID-19 pandemic, the conduct of in-person interviews was considered not feasible due to infection risk of vulnerable population groups and potential non-participation for this reason. Therefore, the preference survey was implemented as a standard electronic or paper survey. All participating patients were encouraged several times to contact the study personnel in case of questions, uncertainties, and technical difficulties, and they were given the possibility to fill the questionnaires on paper. It is possible that some individuals had difficulties in responding to the questions, potentially reflected in the withdrawals and incomplete questionnaires (primarily paper questionnaires) returned by some participants. However, the study personnel and involved experts were contacted relatively infrequently due to questions. Overall, experience of this study showed that such an approach was feasible in the specific population of mPC patients.

Supplementary Figure 2: Participant flow chart for enrolment of metastatic prostate cancer patients and men from the general population into the study.

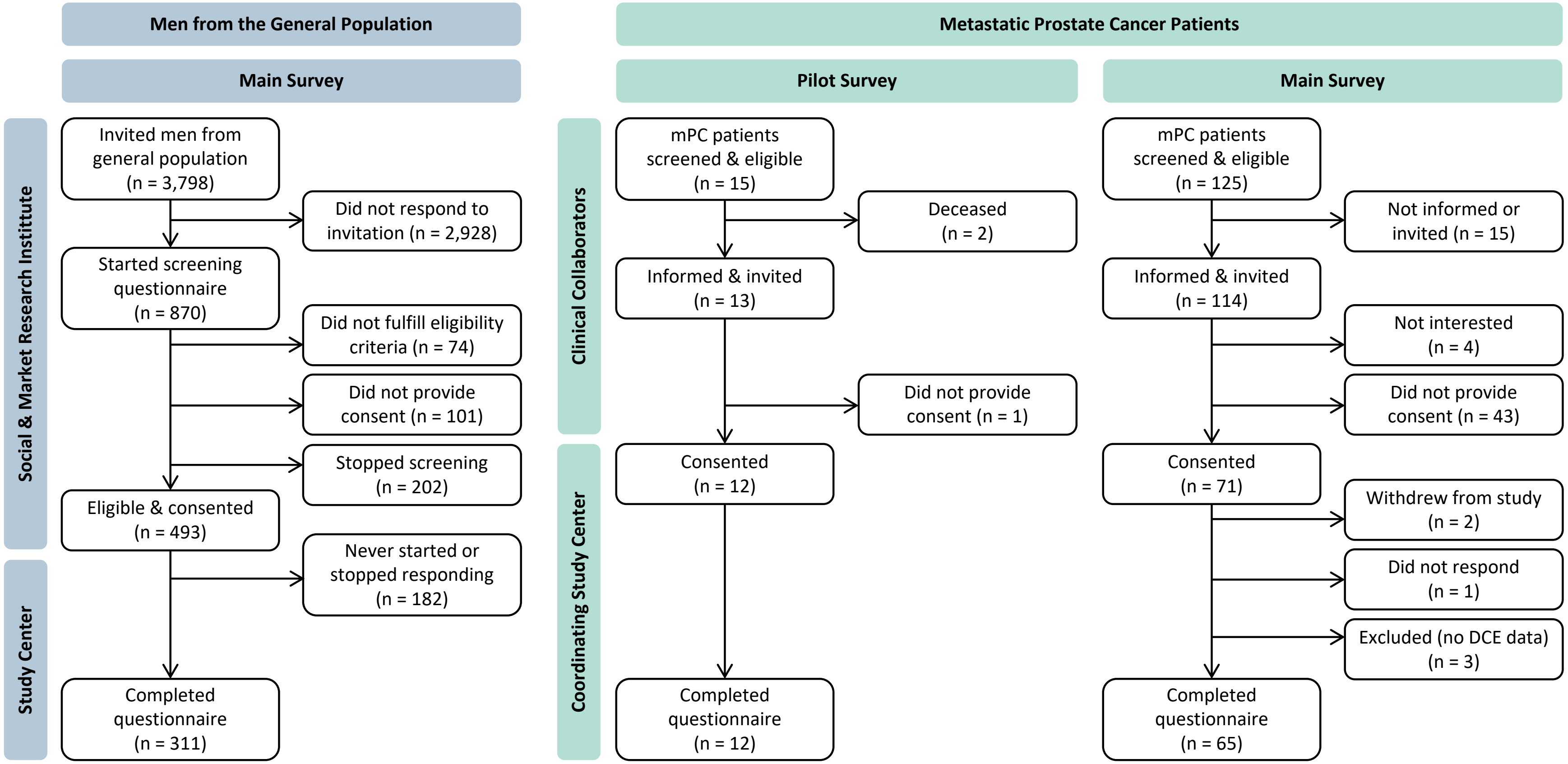

Legend: DCE: discrete choice experiment, mPC: metastatic prostate cancer.

Study Sample and Sample Size Considerations

Planned Recruitment

For the main preference survey, we had aimed to include mPC patients and men from the general population from all language regions of Switzerland (German-, French- and Italian-speaking). The reason for this is that there may be cultural differences between regions that may also affect patients' and the public's perceptions and preferences related to cancer treatment [27,28]. While we were fully able to recruit participants for both population samples in the German- and French-speaking region, we were only able to include Italian-speaking participants for the general population sample. Efforts to recruit mPC patients in the Italian-speaking region were impaired by the COVID-19 pandemic and resulting strain on professional capacity for recruitment. Hence, we refrained from investigating differences in preferences between language regions.

Sample Size

We had planned a sample size of 440 participants for the study, consisting of 140 mPC patients and 300 men from the general population. Sample size calculations for DCEs depend on a large number of factors such as the experimental design, assumptions about the expected parameter estimates, underlying preference heterogeneity, intended comparisons, and statistical models [6,29]. We based our target sample size on the guidance by Orme [30] and de Bekker-Grob et al. [29], insights from a simulation study of optimal sample sizes for DCEs [6], and sample sizes used in other quantitative preference studies conducted in the context of advanced prostate cancer [8]. These recommendations and previous experiences indicated that a reasonable precision would be achieved with sample sizes of more than 100-120 (and up to 300) participants. Hence, we assumed the planned sample sizes to yield sufficient statistical power to derive the required preference estimates, while taking into account our chosen approach and prior assumptions.

While the planned sample size was reached for the general population sample, we were only able to recruit 65 participants for the mPC patient sample. This sample is lower than what we expected at the outset of the project based on our calculations on the number of affected men in the population covered

by the recruiting centers. Meanwhile, the participation rate of 65.4% slightly exceeded what we expected (60%). Since the experimental design of the DCE choice tasks did not change between pilot and main surveys, we included the data gathered during pilot testing in the primary analysis, resulting in a total sample size of 77 mPC patients. To evaluate the appropriateness of this step, we conducted a sensitivity analysis in which we present preference estimates separately for the pilot and main survey participants in the patient sample (Supplementary Table 9).

Supplementary Results

Detailed Study Population Characteristics

Supplementary Table 4: Detailed participant characteristics of included metastatic prostate cancer patients, stratified by pilot and main survey, and men from the general population.

	**Prostate Cancer**		**General Population**	**Overall**
	**Main survey**	**Pilot survey**		
	(N=65)	(N=12)	(N=311)	(N=388)
**Age (years)**				
Mean (SD)	71.5 (8.6)	69.2 (4.6)	63.5 (9.2)	65.0 (9.5)
Median (IQR)	74.0 (65.0 to 78.0)	70.5 (67.0 to 72.2)	64.0 (56.5 to 71.0)	66.0 (58.0 to 73.0)
Range	51 to 86	59 to 75	45 to 80	45 to 86
**Age group**				
45-64 years	16 (24.6%)	2 (16.7%)	156 (50.2%)	174 (44.8%)
65+ years	49 (75.4%)	10 (83.3%)	155 (49.8%)	214 (55.2%)
**Current health status (VAS 1–100)**				
Mean (SD)	72.8 (15.7)	82.1 (7.8)	84.5 (11.3)	82.2 (13.0)
Median (IQR)	75.0 (65.0 to 85.0)	80.0 (75.0 to 90.0)	85.0 (80.0 to 90.0)	85.0 (75.0 to 90.0)
Range	33 to 100	70 to 95	15 to 100	15 to 100
Missing	*0 (0%)*	*0 (0%)*	*49 (15.8%)*	*49 (12.6%)*
**Comorbidity**[a]				
At least one present	37 (56.9%)	9 (75.0%)	146 (46.9%)	192 (49.5%)
Hypertension	18 (48.6%)	6 (66.7%)	96 (65.8%)	120 (62.5%)
Diabetes mellitus	12 (32.4%)	2 (22.2%)	28 (19.2%)	42 (21.9%)
Cardiovascular disease	15 (40.5%)	5 (55.6%)	41 (28.1%)	61 (31.8%)
Chronic respiratory disease	3 (8.1%)	1 (11.1%)	19 (13.0%)	23 (12.0%)
Chronic kidney disease	4 (10.8%)	0 (0.0%)	6 (4.1%)	10 (5.2%)
Chronic liver disease	0 (0.0%)	0 (0.0%)	3 (2.1%)	3 (1.6%)
Other cancer diagnosis	3 (8.1%)	0 (0.0%)	4 (2.7%)	7 (3.6%)

**Smoking status**				
Non-smoker	36 (55.4%)	11 (91.7%)	170 (55.2%)	217 (56.4%)
Non-smoker, previous smoker	24 (36.9%)	1 (8.3%)	83 (26.9%)	108 (28.1%)
Occasional smoker	3 (4.6%)	0 (0.0%)	10 (3.2%)	13 (3.4%)
Regular smoker	2 (3.1%)	0 (0.0%)	45 (14.6%)	47 (12.2%)
Missing	*0 (0%)*	*0 (0%)*	*3 (1.0%)*	*3 (0.8%)*
**Education**				
None or mandatory school	4 (6.2%)	0 (0.0%)	3 (1.0%)	7 (1.8%)
Vocational training or baccalaureate	32 (50.0%)	6 (50.0%)	166 (53.4%)	204 (52.7%)
Higher technical school or college	12 (18.8%)	2 (16.7%)	72 (23.2%)	86 (22.2%)
University degree or doctorate	16 (25.0%)	4 (33.3%)	70 (22.5%)	90 (23.3%)
Missing	*1 (1.5%)*	*0 (0%)*	*0 (0%)*	*1 (0.3%)*
**Employment status**				
Employed or self-employed	13 (20.3%)	2 (16.7%)	139 (44.7%)	154 (39.8%)
Retired	49 (76.6%)	10 (83.3%)	163 (52.4%)	222 (57.4%)
Permanently on sick leave or without work	2 (3.1%)	0 (0.0%)	9 (2.9%)	11 (2.8%)
Missing	*1 (1.5%)*	*0 (0%)*	*0 (0%)*	*1 (0.3%)*
**Income**				
<6,000 CHF	20 (31.2%)	3 (25.0%)	73 (23.5%)	96 (24.8%)
6,000 - 12,000 CHF	26 (40.6%)	6 (50.0%)	165 (53.1%)	197 (50.9%)
>12,000 CHF	13 (20.3%)	1 (8.3%)	48 (15.4%)	62 (16.0%)
No answer	5 (7.8%)	2 (16.7%)	25 (8.0%)	32 (8.3%)
Missing	*1 (1.5%)*	*0 (0%)*	*0 (0%)*	*1 (0.3%)*
**Partnership status**				
In partnership	60 (93.8%)	12 (100.0%)	255 (82.8%)	327 (85.2%)
Missing	*1 (1.5%)*	*0 (0%)*	*3 (1.0%)*	*4 (1.0%)*
**Divorced or widowed**				
Widowed and/or divorced	14 (23.3%)	1 (8.3%)	57 (18.6%)	72 (19.0%)
Missing	*5 (7.7%)*	*0 (0%)*	*5 (1.6%)*	*10 (2.6%)*
**Presence of dependents**				
Having dependents	6 (9.4%)	0 (0.0%)	62 (20.0%)	68 (17.6%)
Missing	*1 (1.5%)*	*0 (0%)*	*1 (0.3%)*	*2 (0.5%)*
**Place of living**				
In the city	11 (16.9%)	1 (8.3%)	69 (22.2%)	81 (20.9%)
In a suburb	21 (32.3%)	4 (33.3%)	100 (32.2%)	125 (32.2%)
On the countryside	33 (50.8%)	7 (58.3%)	142 (45.7%)	182 (46.9%)
**Language region**				
German-speaking	40 (61.5%)	12 (100.0%)	159 (51.1%)	211 (54.4%)
French-speaking	24 (36.9%)	0 (0.0%)	103 (33.1%)	127 (32.7%)
Italian-speaking	1 (1.5%)	0 (0.0%)	49 (15.8%)	50 (12.9%)

**Nationality**				
Swiss	58 (89.2%)	11 (91.7%)	292 (93.9%)	361 (93.0%)
Non-Swiss	7 (10.8%)	1 (8.3%)	19 (6.1%)	27 (7.0%)
**Time since diagnosis (years)**				
Mean (SD)	6.3 (5.2)	9.0 (6.0)	–	–
Median (IQR)	4.5 (2.0 to 8.0)	9.0 (4.0 to 13.2)	–	–
Range	0 to 20	1 to 19	–	–
Missing	*3 (4.6%)*	*0 (0%)*		
**Time since metastasis (years)**				
Mean (SD)	4.0 (3.3)	6.7 (4.5)	–	–
Median (IQR)	3.0 (2.0 to 6.0)	5.0 (4.0 to 10.2)	–	–
Range	0 to 14	1 to 16	–	–
Missing	*5 (7.7%)*	*0 (0%)*		
**Current stage**				
mHSPC	47 (72.3%)	10 (83.3%)	–	–
mCRPC	18 (27.7%)	2 (16.7%)	–	–
**Presence of bone metastases**				
Bone metastases present	46 (70.8%)	8 (66.7%)	–	–
**Ever received treatment for metastatic prostate cancer**				
Ever received treatment	65 (100.0%)	12 (100.0%)	–	–
**Current treatment**[b]				
Currently receiving treatment	63 (96.9%)	11 (91.7%)	–	–
Chemotherapy	9 (14.3%)	1 (9.1%)	–	–
Abiraterone	13 (20.6%)	2 (18.2%)	–	–
Enzalutamide	15 (23.8%)	6 (54.5%)	–	–
Apalutamide	9 (14.3%)	0 (0.0%)	–	–
Darolutamide	1 (1.6%)	0 (0.0%)	–	–
Leuprolide	12 (19.0%)	1 (9.1%)	–	–
Goserelin	5 (7.9%)	7 (63.6%)	–	–
Degarelix	1 (1.6%)	0 (0.0%)	–	–
Hormone therapy (unspecified)	23 (36.5%)	0 (0.0%)	–	–
Lutetium-177 PSMA	6 (9.5%)	1 (9.1%)	–	–
Olaparide	1 (1.6%)	0 (0.0%)	–	–
Metformin	2 (3.2%)	0 (0.0%)	–	–
Radiotherapy	2 (3.2%)	0 (0.0%)	–	–
Orchiectomy	0 (0.0%)	1 (9.1%)	–	–
**Time since beginning of treatment (years)**				
Mean (SD)	2.5 (2.9)	3.5 (3.3)	–	–
Median (IQR)	2.0 (1.0 to 3.0)	4.0 (1.0 to 4.5)	–	–
Range	0 to 13	0 to 11	–	–

Missing	*6 (9.2%)*	*1 (8.3%)*		
**Past treatment**				
Received treatment in the past	31 (47.7%)	8 (66.7%)	–	–
**Adverse effects experience**				
Ever experienced adverse effects	43 (69.4%)	6 (66.7%)	–	–
Missing	*3 (4.6%)*	*3 (25.0%)*		
**Adverse effects experience during current treatment[c]**				
Ever experienced adverse effects during current treatment	35 (56.5%)	5 (55.6%)	–	–
Fatigue	20 (31.7%)	1 (9.1%)	–	–
Hot flushes	11 (17.5%)	4 (36.4%)	–	–
Difficulties with memory or concentration	2 (3.2%)	0 (0.0%)	–	–
Headache	2 (3.2%)	0 (0.0%)	–	–
Vertigo	2 (3.2%)	0 (0.0%)	–	–
Reduced muscle strength	5 (7.9%)	1 (9.1%)	–	–
Hypertension	1 (1.6%)	0 (0.0%)	–	–
Edema	1 (1.6%)	0 (0.0%)	–	–
Dyspnea	1 (1.6%)	0 (0.0%)	–	–
Hyperglycemia or diabetes mellitus	1 (1.6%)	0 (0.0%)	–	–
Nausea	2 (3.2%)	0 (0.0%)	–	–
Weight loss	3 (4.8%)	0 (0.0%)	–	–
Diarrhea	3 (4.8%)	0 (0.0%)	–	–
Sleep disturbances	1 (1.6%)	2 (18.2%)	–	–
Reduced libido	5 (7.9%)	1 (9.1%)	–	–
Erectile dysfunction	1 (1.6%)	0 (0.0%)	–	–
Rash	3 (4.8%)	0 (0.0%)	–	–
Peripheral neuropathy	3 (4.8%)	0 (0.0%)	–	–
Hair or nail loss	1 (1.6%)	1 (9.1%)	–	–
Other	11 (17.5%)	1 (9.1%)	–	–
Missing	*1 (1.6%)*	*2 (18.2%)*		
**Adverse effects experience during past treatment**				
Ever experienced adverse effects during past treatment	18 (64.3%)	1 (16.7%)	–	–
Missing	*37 (56.9%)*	*6 (50.0%)*		
**Pain due to prostate cancer**				
Experienced pain due to prostate cancer in past two weeks	14 (22.2%)	0 (0.0%)	–	–
Missing	*2 (3.1%)*	*0 (0%)*		

**Worst pain level in past 24 hours (VAS 1–10)**				
Mean (SD)	3.1 (1.7)	–	–	–
Median (IQR)	3.0 (2.2 to 3.0)	–	–	–
Range	0 to 7	–	–	–
**Personal or professional experience with cancer**				
Any personal or professional experience with cancer	–	–	236 (76.1%)	–
Missing			*1 (0.3%)*	
**Personal experience with prostate cancer**				
Any personal experience with prostate cancer	–	–	98 (31.6%)	–
Missing			*1 (0.3%)*	
**Personal experience with cancer in general**				
Any personal experience with cancer	–	–	215 (70.3%)	–
Missing			*5 (1.6%)*	
**Professional experience with cancer**				
Any professional experience with cancer	–	–	30 (9.8%)	–
Missing			*4 (1.3%)*	

Legend: CHF: Swiss Francs, IQR: interquartile range, mHSPC: metastatic hormone-sensitive prostate cancer, mCRPC: metastatic castration-resistant prostate cancer, SD: standard deviation, VAS: visual analog scale. [a] For specific comorbidities, reported percentages are calculated based on those reporting at least one comorbidity. [b] For specific treatments, reported percentages are calculated based on those reporting to receive a treatment currently. [c] For specific adverse effects, reported percentages are calculated based on those reporting to experience adverse effects currently.

Assessment of Internal Validity

Supplementary Table 5: Findings from internal validity assessment, based on performance in the dominance test, participant choices, and survey completion time.

	Prostate cancer (N=74)[a]	General population (N=311)[a]	Overall (N=385)[a]
**Correctly responded to the dominance test**	73 (98.6%)	301 (96.8%)	374 (97.1%)
**Consistently chose the same alternative (A (left) or B (right))**	3 (4.1%)	4 (1.3%)	7 (1.8%)
**Based all choices on overall survival attribute**	5 (6.8%)	7 (2.3%)	12 (3.1%)
**Based all choices on any of the harm attributes**	0 (0.0%)	0 (0.0%)	0 (0.0%)
**Took less than 15 minutes to complete the preference survey**	2 (2.7%)	75 (24.1%)	77 (20.0%)
**Fulfilled all criteria**	66 (89.2%)	225 (72.3%)	291 (75.6%)

Legend: [a] The total N represents the number of fully completed questionnaires (three metastatic prostate cancer patients did not complete the discrete choice experiment).

Supplementary Figure 3: Time required by participants to fill the discrete choice experiment (DCE) choice tasks, by individual choice task (panel **A**) and by sequence of display (panel **B**; responses without timing information (e.g., paper questionnaires) were omitted). Points represent median time in seconds and whiskers represent interquartile ranges. The twelfth DCE was the dominance test, which included a clearly dominant alternative.

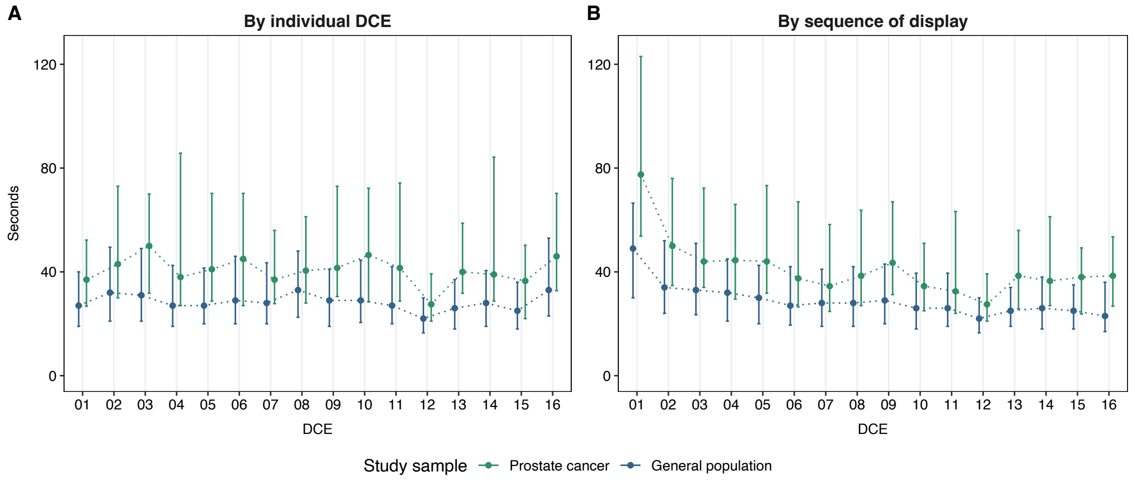

Supplementary Figure 4: Evaluation of the experiences of study participants with the discrete choice experiment (DCE) choice tasks (responses from pilot testing with metastatic prostate cancer patients were omitted due to slightly amended wording of statements).

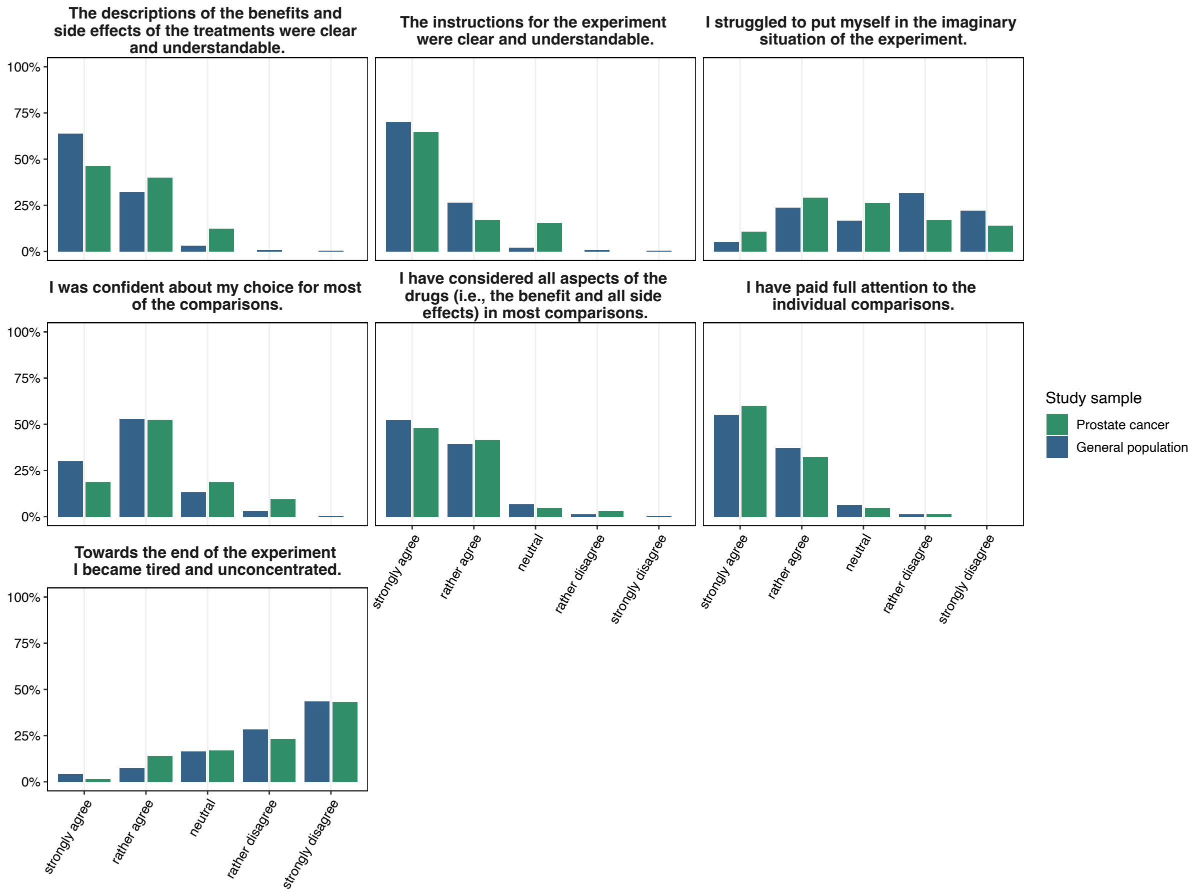

Primary Analysis

Supplementary Table 6: Preference weights derived from primary analysis. Preference weights with 95% confidence intervals were estimated for the overall study population and separately for metastatic prostate cancer patients and men from the general population using dummy-coded mixed logit models with overall survival (OS) as a random parameter (negative values represent a preference for averting the outcome).

	**Prostate Cancer** (N=77)		**General Population** (N=311)		**Test for difference[a]**
	Coeff. (95% CI)	p-value	Coeff. (95% CI)	p-value	p-value
**Overall survival**					
1-year OS benefit	1.201 (0.810 to 1.592)	<0.001	0.590 (0.440 to 0.740)	<0.001	0.004
**Diarrhea**					
None	Ref.		Ref.		
Mild	-0.352 (-0.608 to -0.096)	0.007	-0.171 (-0.288 to -0.054)	0.004	0.208
Moderate	-0.717 (-0.967 to -0.466)	<0.001	-0.600 (-0.713 to -0.487)	<0.001	0.405
**Fatigue**					
None	Ref.		Ref.		
Mild	-0.081 (-0.341 to 0.179)	0.541	-0.202 (-0.317 to -0.088)	0.001	0.401
Moderate	-0.608 (-0.895 to -0.321)	<0.001	-0.751 (-0.882 to -0.619)	<0.001	0.377
**Peripheral neuropathy**					
None	Ref.		Ref.		
Mild	0.044 (-0.234 to 0.322)	0.757	0.089 (-0.036 to 0.214)	0.16	0.772
Moderate	-0.258 (-0.584 to 0.069)	0.122	-0.186 (-0.321 to -0.051)	0.007	0.692
**Fracture**					
None	Ref.		Ref.		
Moderate	-0.335 (-0.617 to -0.052)	0.020	-0.443 (-0.570 to -0.316)	<0.001	0.492

Severe	-1.768 (-2.165 to -1.370)	<0.001	-1.549 (-1.723 to -1.375)	<0.001	0.323
**Ischemic heart disease**					
None	Ref.		Ref.		
Moderate	-0.728 (-1.033 to -0.423)	<0.001	-0.657 (-0.791 to -0.524)	<0.001	0.676
Severe	-2.317 (-2.769 to -1.865)	<0.001	-2.105 (-2.289 to -1.921)	<0.001	0.395
Very severe	-3.634 (-4.191 to -3.077)	<0.001	-3.356 (-3.592 to -3.119)	<0.001	0.368
**Rash**					
None	Ref.		Ref.		
Mild	-0.434 (-0.634 to -0.234)	<0.001	-0.233 (-0.331 to -0.135)	<0.001	0.076
Moderate	-0.658 (-0.948 to -0.369)	<0.001	-0.510 (-0.640 to -0.379)	<0.001	0.358
**Standard deviations (SD)[b]**					
1-year OS benefit	1.308 (0.938 to 1.678)	<0.001	1.039 (0.891 to 1.186)	<0.001	0.186
**Model details**					
Log-likelihood	-581.7		-2548.4		
AIC	1193.5		5126.9		
BIC	1269.7		5224.6		

Legend: AIC: Akaike information criterion, BIC: Bayesian information criterion, Coeff.: coefficient (preference weight), CI: confidence interval, OS: overall survival, Ref.: reference. [a] P-values calculated using z-tests for a difference between prostate cancer and general population sample participants, estimated based on separate mixed multinomial logit models. [b] Standard deviations derived from mixed multinomial logit models are a measure for the preference heterogeneity between individuals in the sample.

Sensitivity Analyses

Supplementary Table 7: Preference weights (and 95% confidence intervals) for metastatic prostate cancer patients, derived from sensitivity analyses based on exclusion of participants failing the internal validity assessment.

**Prostate cancer patients**	**Completed in ≥15 min** (N=75)		**Passed dominance test** (N=73)		**Considered all alternatives** (N=74)		**Choices based on several attributes** (N=72)		**Fulfilled all validity criteria** (N=66)	
	Coeff. (95% CI)	p-value	Coeff. (95% CI)	p-value	Coeff. (95% CI)	p-value	Coeff. (95% CI)	p-value	Coeff. (95% CI)	p-value
**Overall survival**										
1-year OS benefit (continuous)	1.203 (0.805 to 1.601)	<0.001	1.251 (0.881 to 1.621)	<0.001	1.196 (0.804 to 1.588)	<0.001	0.926 (0.613 to 1.240)	<0.001	1.011 (0.703 to 1.319)	<0.001
**Diarrhea**										
None	Ref.		Ref.		Ref.		Ref.		Ref.	
Mild	-0.388 (-0.650 to -0.125)	0.004	-0.323 (-0.582 to -0.063)	0.015	-0.345 (-0.601 to -0.089)	0.008	-0.321 (-0.578 to -0.064)	0.014	-0.324 (-0.594 to -0.054)	0.019
Moderate	-0.721 (-0.974 to -0.468)	<0.001	-0.703 (-0.955 to -0.450)	<0.001	-0.714 (-0.965 to -0.464)	<0.001	-0.694 (-0.942 to -0.447)	<0.001	-0.690 (-0.944 to -0.436)	<0.001
**Fatigue**										
None	Ref.		Ref.		Ref.		Ref.		Ref.	
Mild	-0.045 (-0.308 to 0.218)	0.735	-0.069 (-0.333 to 0.196)	0.610	-0.076 (-0.336 to 0.183)	0.564	-0.025 (-0.282 to 0.232)	0.850	0.022 (-0.247 to 0.292)	0.871
Moderate	-0.552 (-0.841 to -0.263)	<0.001	-0.625 (-0.916 to -0.335)	<0.001	-0.615 (-0.903 to -0.327)	<0.001	-0.590 (-0.874 to -0.305)	<0.001	-0.564 (-0.858 to -0.270)	<0.001
**Peripheral neuropathy**										
None	Ref.		Ref.		Ref.		Ref.		Ref.	
Mild	0.070 (-0.214 to 0.354)	0.630	0.042 (-0.238 to 0.321)	0.770	0.038 (-0.240 to 0.316)	0.791	0.014 (-0.258 to 0.286)	0.919	0.036 (-0.246 to 0.318)	0.804

Moderate	-0.227 (-0.553 to 0.100)	0.174	-0.257 (-0.593 to 0.078)	0.133	-0.255 (-0.580 to 0.071)	0.125	-0.182 (-0.497 to 0.134)	0.259	-0.169 (-0.503 to 0.164)	0.320
**Fracture**										
None	Ref.		Ref.		Ref.		Ref.		Ref.	
Moderate	-0.321 (-0.605 to -0.036)	0.027	-0.300 (-0.587 to -0.014)	0.040	-0.339 (-0.622 to -0.056)	0.019	-0.293 (-0.571 to -0.015)	0.039	-0.247 (-0.533 to 0.039)	0.091
Severe	-1.725 (-2.125 to -1.325)	<0.001	-1.753 (-2.153 to -1.353)	<0.001	-1.770 (-2.167 to -1.373)	<0.001	-1.661 (-2.045 to -1.278)	<0.001	-1.627 (-2.020 to -1.234)	<0.001
**Ischemic heart disease**										
None	Ref.		Ref.		Ref.		Ref.		Ref.	
Moderate	-0.739 (-1.050 to -0.428)	<0.001	-0.713 (-1.020 to -0.406)	<0.001	-0.727 (-1.032 to -0.423)	<0.001	-0.641 (-0.942 to -0.340)	<0.001	-0.649 (-0.962 to -0.336)	<0.001
Severe	-2.290 (-2.743 to -1.837)	<0.001	-2.316 (-2.776 to -1.856)	<0.001	-2.311 (-2.763 to -1.860)	<0.001	-2.202 (-2.634 to -1.771)	<0.001	-2.211 (-2.660 to -1.761)	<0.001
Very severe	-3.605 (-4.164 to -3.045)	<0.001	-3.643 (-4.206 to -3.080)	<0.001	-3.640 (-4.197 to -3.084)	<0.001	-3.480 (-4.013 to -2.948)	<0.001	-3.512 (-4.062 to -2.963)	<0.001
**Rash**										
None	Ref.		Ref.		Ref.		Ref.		Ref.	
Mild	-0.406 (-0.608 to -0.204)	<0.001	-0.426 (-0.625 to -0.226)	<0.001	-0.422 (-0.622 to -0.221)	<0.001	-0.414 (-0.615 to -0.213)	<0.001	-0.376 (-0.580 to -0.172)	<0.001
Moderate	-0.652 (-0.946 to -0.358)	<0.001	-0.656 (-0.948 to -0.364)	<0.001	-0.657 (-0.946 to -0.368)	<0.001	-0.617 (-0.904 to -0.329)	<0.001	-0.611 (-0.907 to -0.314)	<0.001
**Standard deviations (SD)[a]**										
1-year OS benefit	1.346 (0.955 to 1.737)	<0.001	1.141 (0.796 to 1.486)	<0.001	1.300 (0.933 to 1.668)	<0.001	0.895 (0.593 to 1.196)	<0.001	0.746 (0.471 to 1.022)	<0.001
**Model details**										
Log-likelihood	-566.2		-569.3		-579.3		-550.7		-519.0	

AIC	1162.5	1168.5	1188.6	1131.4	1068.0
BIC	1238.2	1244.5	1264.7	1206.6	1142.4

Legend: AIC: Akaike information criterion, BIC: Bayesian information criterion, Coeff.: coefficient (preference weight), CI: confidence interval, OS: overall survival, Ref.: reference. [a] Standard deviations derived from mixed multinomial logit models are a measure for the preference heterogeneity between individuals in the sample.

Supplementary Table 8: Preference weights (and 95% confidence intervals) for men from the general population, derived from sensitivity analyses based on exclusion of participants failing the internal validity assessment.

Men from general population	Completed in ≥15 min (N=236)		Passed dominance test (N=301)		Considered all alternatives (N=307)		Choices based on several attributes (N=304)		Fulfilled all validity criteria (N=225)	
	Coeff. (95% CI)	p-value	Coeff. (95% CI)	p-value	Coeff. (95% CI)	p-value	Coeff. (95% CI)	p-value	Coeff. (95% CI)	p-value
**Overall survival**										
1-year OS benefit (continuous)	0.694 (0.510 to 0.877)	<0.001	0.698 (0.541 to 0.854)	<0.001	0.606 (0.451 to 0.761)	<0.001	0.495 (0.358 to 0.632)	<0.001	0.702 (0.523 to 0.882)	<0.001
**Diarrhea**										
None	Ref.		Ref.		Ref.		Ref.		Ref.	
Mild	-0.174 (-0.312 to -0.037)	0.013	-0.174 (-0.296 to -0.053)	0.005	-0.177 (-0.297 to -0.058)	0.004	-0.159 (-0.278 to -0.041)	0.008	-0.171 (-0.314 to -0.028)	0.019
Moderate	-0.648 (-0.781 to -0.514)	<0.001	-0.645 (-0.763 to -0.527)	<0.001	-0.622 (-0.737 to -0.507)	<0.001	-0.592 (-0.704 to -0.479)	<0.001	-0.683 (-0.822 to -0.543)	<0.001
**Fatigue**										
None	Ref.		Ref.		Ref.		Ref.		Ref.	
Mild	-0.204 (-0.339 to -0.069)	0.003	-0.240 (-0.360 to -0.121)	<0.001	-0.214 (-0.331 to -0.098)	<0.001	-0.179 (-0.294 to -0.064)	0.002	-0.210 (-0.352 to -0.069)	0.004

Moderate	-0.778 (-0.934 to -0.623)	<0.001	-0.846 (-0.984 to -0.707)	<0.001	-0.779 (-0.913 to -0.645)	<0.001	-0.745 (-0.877 to -0.613)	<0.001	-0.848 (-1.011 to -0.686)	<0.001
**Peripheral neuropathy**										
None	Ref.		Ref.		Ref.		Ref.		Ref.	
Mild	0.040 (-0.106 to 0.186)	0.592	0.073 (-0.056 to 0.201)	0.267	0.086 (-0.041 to 0.213)	0.187	0.071 (-0.054 to 0.195)	0.267	-0.000 (-0.150 to 0.150)	0.998
Moderate	-0.238 (-0.400 to -0.077)	0.004	-0.270 (-0.415 to -0.125)	<0.001	-0.207 (-0.345 to -0.069)	0.003	-0.164 (-0.298 to -0.029)	0.017	-0.281 (-0.452 to -0.111)	0.001
**Fracture**										
None	Ref.		Ref.		Ref.		Ref.		Ref.	
Moderate	-0.464 (-0.617 to -0.311)	<0.001	-0.483 (-0.617 to -0.348)	<0.001	-0.467 (-0.597 to -0.337)	<0.001	-0.437 (-0.564 to -0.310)	<0.001	-0.499 (-0.659 to -0.339)	<0.001
Severe	-1.699 (-1.911 to -1.488)	<0.001	-1.660 (-1.845 to -1.474)	<0.001	-1.609 (-1.788 to -1.430)	<0.001	-1.525 (-1.698 to -1.352)	<0.001	-1.782 (-2.004 to -1.559)	<0.001
**Ischemic heart disease**										
None	Ref.		Ref.		Ref.		Ref.		Ref.	
Moderate	-0.643 (-0.800 to -0.485)	<0.001	-0.680 (-0.818 to -0.541)	<0.001	-0.680 (-0.815 to -0.544)	<0.001	-0.639 (-0.772 to -0.506)	<0.001	-0.651 (-0.814 to -0.489)	<0.001
Severe	-2.279 (-2.503 to -2.055)	<0.001	-2.283 (-2.483 to -2.084)	<0.001	-2.178 (-2.368 to -1.988)	<0.001	-2.077 (-2.259 to -1.895)	<0.001	-2.419 (-2.658 to -2.181)	<0.001
Very severe	-3.631 (-3.923 to -3.340)	<0.001	-3.637 (-3.893 to -3.381)	<0.001	-3.471 (-3.715 to -3.227)	<0.001	-3.319 (-3.553 to -3.085)	<0.001	-3.852 (-4.163 to -3.541)	<0.001
**Rash**										
None	Ref.		Ref.		Ref.		Ref.		Ref.	
Mild	-0.228 (-0.342 to -0.114)	<0.001	-0.250 (-0.351 to -0.149)	<0.001	-0.240 (-0.340 to -0.140)	<0.001	-0.220 (-0.319 to -0.121)	<0.001	-0.240 (-0.358 to -0.122)	<0.001
Moderate	-0.619 (-0.774 to -0.463)	<0.001	-0.532 (-0.668 to -0.396)	<0.001	-0.532 (-0.666 to -0.399)	<0.001	-0.501 (-0.632 to -0.371)	<0.001	-0.658 (-0.820 to -0.496)	<0.001

**Standard deviations (SD)**[a]										
1-year OS benefit	1.096 (0.922 to 1.271)	<0.001	1.040 (0.889 to 1.192)	<0.001	1.068 (0.918 to 1.218)	<0.001	0.896 (0.759 to 1.033)	<0.001	0.998 (0.829 to 1.168)	<0.001
**Model details**										
Log-likelihood	-1873.1		-2391.5		-2482.8		-2494.6		-1739.7	
AIC	3776.2		4812.9		4995.6		5019.3		3509.4	
BIC	3869.8		4910.1		5093.1		5116.6		3602.2	

Legend: AIC: Akaike information criterion, BIC: Bayesian information criterion, Coeff.: coefficient (preference weight), CI: confidence interval, OS: overall survival, Ref.: reference. [a] Standard deviations derived from mixed multinomial logit models are a measure for the preference heterogeneity between individuals in the sample.

Supplementary Table 9: Preference weights (and 95% confidence intervals) for metastatic prostate cancer patients, derived from sensitivity analyses based on the pilot and main preference survey.

	**Pilot & main survey** (N=77)		**Pilot survey** (N=12)		**Main study survey** (N=65)		**Test for difference**[a]
	Coeff. (95% CI)	p-value	Coeff. (95% CI)	p-value	Coeff. (95% CI)	p-value	
**Overall survival**							
1-year OS benefit	1.201 (0.810 to 1.592)	<0.001	1.321 (0.504 to 2.137)	0.002	1.253 (0.791 to 1.715)	<0.001	0.888
**Diarrhea**							
None	Ref.		Ref.		Ref.		
Mild	-0.352 (-0.608 to -0.096)	0.007	-0.828 (-1.828 to 0.173)	0.105	-0.350 (-0.628 to -0.072)	0.014	0.368
Moderate	-0.717 (-0.967 to -0.466)	<0.001	-0.811 (-1.541 to -0.081)	0.029	-0.748 (-1.025 to -0.471)	<0.001	0.875
**Fatigue**							
None	Ref.		Ref.		Ref.		

Mild	-0.081 (-0.341 to 0.179)	0.541	0.732 (-0.279 to 1.743)	0.156	-0.146 (-0.431 to 0.140)	0.317	0.102
Moderate	-0.608 (-0.895 to -0.321)	<0.001	-0.062 (-0.818 to 0.693)	0.872	-0.689 (-1.011 to -0.368)	<0.001	0.135
**Peripheral neuropathy**							
None	Ref.		Ref.		Ref.		
Mild	0.044 (-0.234 to 0.322)	0.757	0.925 (-0.185 to 2.036)	0.102	-0.027 (-0.331 to 0.277)	0.862	0.105
Moderate	-0.258 (-0.584 to 0.069)	0.122	0.129 (-0.690 to 0.948)	0.758	-0.296 (-0.662 to 0.070)	0.113	0.353
**Fracture**							
None	Ref.		Ref.		Ref.		
Moderate	-0.335 (-0.617 to -0.052)	0.020	0.035 (-0.684 to 0.755)	0.924	-0.390 (-0.706 to -0.073)	0.016	0.290
Severe	-1.768 (-2.165 to -1.370)	<0.001	-1.795 (-2.774 to -0.816)	<0.001	-1.818 (-2.262 to -1.374)	<0.001	0.967
**Ischemic heart disease**							
None	Ref.		Ref.		Ref.		
Moderate	-0.728 (-1.033 to -0.423)	<0.001	-1.612 (-2.839 to -0.384)	0.010	-0.669 (-1.000 to -0.338)	<0.001	0.146
Severe	-2.317 (-2.769 to -1.865)	<0.001	-2.146 (-3.206 to -1.086)	<0.001	-2.415 (-2.926 to -1.905)	<0.001	0.654
Very severe	-3.634 (-4.191 to -3.077)	<0.001	-3.912 (-5.344 to -2.481)	<0.001	-3.672 (-4.294 to -3.049)	<0.001	0.763
**Rash**							
None	Ref.		Ref.		Ref.		
Mild	-0.434 (-0.634 to -0.234)	<0.001	-0.174 (-0.688 to 0.341)	0.508	-0.506 (-0.728 to -0.285)	<0.001	0.244
Moderate	-0.658 (-0.948 to -0.369)	<0.001	-1.243 (-2.254 to -0.232)	0.016	-0.629 (-0.946 to -0.313)	<0.001	0.256
**Standard deviations (SD)[b]**							
1-year OS benefit	1.308 (0.938 to 1.678)	<0.001	0.802 (0.117 to 1.486)	0.022	1.437 (1.003 to 1.871)	<0.001	0.124
**Model details**							
Log-likelihood	-581.7		-90.1		-484.6		
AIC	1193.5		210.3		999.2		
BIC	1269.7		259.1		1072.8		

Legend: AIC: Akaike information criterion, BIC: Bayesian information criterion, Coeff.: coefficient (preference weight), CI: confidence interval, OS: overall survival, Ref.: reference. [a] P-values calculated using z-tests for a difference between pilot and main study sample of metastatic prostate cancer patients, estimated based on separate mixed multinomial logit models. Please note the low number of participants in the pilot sample and the relevant preference heterogeneity when interpreting the results. [b] Standard deviations derived from mixed multinomial logit models are a measure for the preference heterogeneity between individuals in the sample.

Supplementary Table 10: Preference weights (and 95% confidence intervals) for metastatic prostate cancer patients, derived from sensitivity analyses based on alternative models.

**Prostate cancer patients**	**Mixed multinomial logit model** (primary analysis; OS as random parameter; N=77)		**Mixed multinomial logit model** (all attributes as random parameters; N=77)		**Multinomial logit model** (OS as continuous outcome; N=77)		**Multinomial logit model** (OS as categorical outcome; N=77)	
	Coeff. (95% CI)	p-value	Coeff. (95% CI)	p-value	Coeff. (95% CI)	p-value	Coeff. (95% CI)	p-value
**Overall survival**								
1-year OS benefit (continuous)	1.201 (0.810 to 1.592)	<0.001	1.557 (1.081 to 2.033)	<0.001	0.850 (0.654 to 1.046)	<0.001		
4.5 years (categorical)							Ref.	
5 years (categorical)							0.721 (0.488 to 0.955)	<0.001
6 years (categorical)							1.283 (0.989 to 1.576)	<0.001
**Diarrhea**								
None	Ref.		Ref.		Ref.		Ref.	
Mild	-0.352 (-0.608 to -0.096)	0.007	-0.427 (-0.765 to -0.090)	0.013	-0.259 (-0.498 to -0.020)	0.034	-0.250 (-0.486 to -0.015)	0.037
Moderate	-0.717 (-0.967 to -0.466)	<0.001	-0.777 (-1.096 to -0.458)	<0.001	-0.520 (-0.738 to -0.302)	<0.001	-0.558 (-0.789 to -0.326)	<0.001
**Fatigue**								
None	Ref.		Ref.		Ref.		Ref.	
Mild	-0.081 (-0.341 to 0.179)	0.541	-0.166 (-0.492 to 0.160)	0.319	0.033 (-0.202 to 0.268)	0.782	0.054 (-0.177 to 0.285)	0.646

Moderate	-0.608 (-0.895 to -0.321)	<0.001	-0.789 (-1.169 to -0.408)	<0.001	-0.445 (-0.702 to -0.187)	0.001	-0.573 (-0.854 to -0.292)	<0.001
**Peripheral neuropathy**								
None	Ref.		Ref.		Ref.		Ref.	
Mild	0.044 (-0.234 to 0.322)	0.757	-0.013 (-0.339 to 0.313)	0.936	0.097 (-0.149 to 0.343)	0.440	0.089 (-0.151 to 0.330)	0.467
Moderate	-0.258 (-0.584 to 0.069)	0.122	-0.410 (-0.826 to 0.006)	0.053	-0.026 (-0.314 to 0.261)	0.857	-0.036 (-0.310 to 0.238)	0.798
**Fracture**								
None	Ref.		Ref.		Ref.		Ref.	
Moderate	-0.335 (-0.617 to -0.052)	0.020	-0.321 (-0.674 to 0.031)	0.074	-0.065 (-0.308 to 0.177)	0.596	-0.157 (-0.419 to 0.105)	0.240
Severe	-1.768 (-2.165 to -1.370)	<0.001	-2.233 (-2.801 to -1.665)	<0.001	-1.198 (-1.507 to -0.889)	<0.001	-1.261 (-1.570 to -0.952)	<0.001
**Ischemic heart disease**								
None	Ref.		Ref.		Ref.		Ref.	
Moderate	-0.728 (-1.033 to -0.423)	<0.001	-0.881 (-1.237 to -0.526)	<0.001	-0.477 (-0.749 to -0.204)	0.001	-0.469 (-0.735 to -0.203)	0.001
Severe	-2.317 (-2.769 to -1.865)	<0.001	-2.878 (-3.499 to -2.258)	<0.001	-1.721 (-2.078 to -1.365)	<0.001	-1.726 (-2.074 to -1.379)	<0.001
Very severe	-3.634 (-4.191 to -3.077)	<0.001	-4.768 (-5.685 to -3.851)	<0.001	-2.797 (-3.216 to -2.379)	<0.001	-2.883 (-3.302 to -2.464)	<0.001
**Rash**								
None	Ref.		Ref.		Ref.		Ref.	
Mild	-0.434 (-0.634 to -0.234)	<0.001	-0.558 (-0.801 to -0.316)	<0.001	-0.344 (-0.522 to -0.165)	<0.001	-0.376 (-0.559 to -0.192)	<0.001
Moderate	-0.658 (-0.948 to -0.369)	<0.001	-0.744 (-1.107 to -0.381)	<0.001	-0.416 (-0.670 to -0.162)	0.001	-0.488 (-0.746 to -0.231)	<0.001
**Standard deviations (SD)[a]**								
1-year OS benefit	1.308 (0.938 to 1.678)	<0.001	1.717 (1.229 to 2.205)	<0.001				
Mild diarrhea			0.391 (-0.273 to 1.055)	0.249				
Moderate diarrhea			0.562 (0.165 to 0.959)	0.006				
Mild fatigue			0.461 (0.057 to 0.866)	0.025				
Moderate fatigue			0.773 (0.304 to 1.242)	0.001				
Mild sensory disturbances			0.017 (-0.389 to 0.424)	0.934				
Moderate sensory disturbances			0.664 (0.078 to 1.250)	0.026				

Moderate fracture			0.538 (0.122 to 0.955)	0.011				
Severe fracture			0.971 (0.568 to 1.373)	<0.001				
Moderate cardiovascular disease			0.903 (0.194 to 1.612)	0.013				
Severe cardiovascular disease			0.092 (-0.418 to 0.602)	0.724				
Very severe cardiovascular disease			0.588 (-0.023 to 1.200)	0.059				
Mild rash			0.076 (-0.389 to 0.541)	0.750				
Moderate rash			0.347 (-0.259 to 0.954)	0.262				
**Model details**								
Log-likelihood	-581.7		-569.2		-618.8		-614.8	
AIC	1193.5		1194.4		1265.6		1259.6	
BIC	1269.7		1336.6		1336.7		1335.8	

Legend: AIC: Akaike information criterion, BIC: Bayesian information criterion, Coeff.: coefficient (preference weight), CI: confidence interval, OS: overall survival, Ref.: reference. [a] Standard deviations derived from mixed multinomial logit models are a measure for the preference heterogeneity between individuals in the sample.

Supplementary Table 11: Preference weights (and 95% confidence intervals) for men from the general population, derived from sensitivity analyses based on alternative models.

Men from general population	**Mixed multinomial logit model** (primary analysis; OS as random parameter; N=311)		**Mixed multinomial logit model** (all attributes as random parameters; N=311)		**Multinomial logit model** (OS as continuous outcome; N=311)		**Multinomial logit model** (OS as categorical outcome; N=311)	
	Coeff. (95% CI)	p-value	Coeff. (95% CI)	p-value	Coeff. (95% CI)	p-value	Coeff. (95% CI)	p-value
**Overall survival**								
1-year OS benefit (continuous)	0.590 (0.440 to 0.740)	<0.001	0.647 (0.479 to 0.816)	<0.001	0.439 (0.357 to 0.520)	<0.001		
4.5 years (categorical)							Ref.	
5 years (categorical)							0.507 (0.399 to 0.615)	<0.001

6 years (categorical)							0.679 (0.554 to 0.803)	<0.001
**Diarrhea**								
None	Ref.		Ref.		Ref.		Ref.	
Mild	-0.171 (-0.288 to -0.054)	0.004	-0.190 (-0.318 to -0.061)	0.004	-0.125 (-0.237 to -0.012)	0.030	-0.149 (-0.262 to -0.036)	0.010
Moderate	-0.600 (-0.713 to -0.487)	<0.001	-0.667 (-0.802 to -0.531)	<0.001	-0.462 (-0.563 to -0.360)	<0.001	-0.523 (-0.630 to -0.416)	<0.001
**Fatigue**								
None	Ref.		Ref.		Ref.		Ref.	
Mild	-0.202 (-0.317 to -0.088)	0.001	-0.272 (-0.402 to -0.143)	<0.001	-0.090 (-0.195 to 0.015)	0.094	-0.069 (-0.174 to 0.036)	0.198
Moderate	-0.751 (-0.882 to -0.619)	<0.001	-0.852 (-1.011 to -0.694)	<0.001	-0.610 (-0.731 to -0.489)	<0.001	-0.698 (-0.824 to -0.572)	<0.001
**Peripheral neuropathy**								
None	Ref.		Ref.		Ref.		Ref.	
Mild	0.089 (-0.036 to 0.214)	0.162	0.062 (-0.074 to 0.198)	0.373	0.068 (-0.045 to 0.181)	0.239	0.069 (-0.046 to 0.184)	0.238
Moderate	-0.186 (-0.321 to -0.051)	0.007	-0.240 (-0.390 to -0.090)	0.002	-0.009 (-0.132 to 0.114)	0.889	-0.026 (-0.147 to 0.094)	0.668
**Fracture**								
None	Ref.		Ref.		Ref.		Ref.	
Moderate	-0.443 (-0.570 to -0.316)	<0.001	-0.513 (-0.656 to -0.371)	<0.001	-0.245 (-0.357 to -0.132)	<0.001	-0.326 (-0.445 to -0.207)	<0.001
Severe	-1.549 (-1.723 to -1.375)	<0.001	-1.847 (-2.085 to -1.610)	<0.001	-1.143 (-1.287 to -0.999)	<0.001	-1.233 (-1.380 to -1.086)	<0.001
**Ischemic heart disease**								
None	Ref.		Ref.		Ref.		Ref.	
Moderate	-0.657 (-0.791 to -0.524)	<0.001	-0.754 (-0.908 to -0.600)	<0.001	-0.468 (-0.589 to -0.346)	<0.001	-0.501 (-0.623 to -0.378)	<0.001
Severe	-2.105 (-2.289 to -1.921)	<0.001	-2.395 (-2.626 to -2.164)	<0.001	-1.669 (-1.820 to -1.518)	<0.001	-1.734 (-1.889 to -1.580)	<0.001
Very severe	-3.356 (-3.592 to -3.119)	<0.001	-4.157 (-4.535 to -3.778)	<0.001	-2.744 (-2.931 to -2.557)	<0.001	-2.840 (-3.031 to -2.650)	<0.001
**Rash**								
None	Ref.		Ref.		Ref.		Ref.	
Mild	-0.233 (-0.331 to -0.135)	<0.001	-0.266 (-0.374 to -0.157)	<0.001	-0.162 (-0.251 to -0.072)	<0.001	-0.193 (-0.284 to -0.102)	<0.001
Moderate	-0.510 (-0.640 to -0.379)	<0.001	-0.560 (-0.708 to -0.411)	<0.001	-0.337 (-0.455 to -0.220)	<0.001	-0.423 (-0.545 to -0.301)	<0.001

**Standard deviations (SD)[a]**								
1-year OS benefit	1.039 (0.891 to 1.186)	<0.001	1.203 (1.026 to 1.380)	<0.001				
Mild diarrhea			0.041 (-0.219 to 0.301)	0.757				
Moderate diarrhea			0.386 (0.152 to 0.621)	0.001				
Mild fatigue			0.017 (-0.275 to 0.309)	0.908				
Moderate fatigue			0.501 (0.271 to 0.731)	<0.001				
Mild sensory disturbances			0.122 (-0.169 to 0.413)	0.410				
Moderate sensory disturbances			0.025 (-0.659 to 0.710)	0.942				
Moderate fracture			0.053 (-0.216 to 0.321)	0.700				
Severe fracture			0.813 (0.592 to 1.033)	<0.001				
Moderate cardiovascular disease			1.280 (0.970 to 1.589)	<0.001				
Severe cardiovascular disease			0.363 (0.101 to 0.626)	0.007				
Very severe cardiovascular disease			0.524 (0.210 to 0.838)	0.001				
Mild rash			0.058 (-0.144 to 0.259)	0.573				
Moderate rash			0.108 (-0.160 to 0.376)	0.429				
**Model details**								
Log-likelihood	-2548.4		-2510.5		-2664.0		-2647.5	
AIC	5126.9		5077.0		5356.0		5324.9	
BIC	5224.6		5259.3		5447.2		5422.6	

Legend: AIC: Akaike information criterion, BIC: Bayesian information criterion, Coeff.: coefficient (preference weight), CI: confidence interval, OS: overall survival, Ref.: reference. [a] Standard deviations derived from mixed multinomial logit models are a measure for the preference heterogeneity between individuals in the sample.

Supplementary Table 12: Preference weights (and 95% confidence intervals) derived from sensitivity analyses using a 1:1 propensity-score matched subsample of study participants for comparison between metastatic prostate cancer (mPC) patients and men from the general population. Propensity score matching was performed

using nearest neighbour matching with a caliper of 0.25, based on age, current health status, presence of comorbidities, smoking status, education, employment, and partnership status, implemented in the *MatchIt* R package (v4.4.0). 64 mPC patients and 64 men from the general population were used in the analysis.

	Prostate Cancer (N=64)		General Population (N=64)		Test for difference[a]
	Coeff. (95% CI)	p-value	Coeff. (95% CI)	p-value	p-value
**Overall survival**					
1-year OS benefit	1.250 (0.785 to 1.715)	<0.001	0.462 (0.082 to 0.842)	0.017	0.010
**Diarrhea**					
None	Ref.		Ref.		
Mild	-0.369 (-0.655 to -0.084)	0.011	-0.370 (-0.640 to -0.100)	0.007	0.998
Moderate	-0.715 (-0.994 to -0.436)	<0.001	-0.623 (-0.873 to -0.372)	<0.001	0.631
**Fatigue**					
None	Ref.		Ref.		
Mild	-0.099 (-0.390 to 0.191)	0.503	-0.047 (-0.303 to 0.209)	0.719	0.790
Moderate	-0.637 (-0.956 to -0.318)	<0.001	-0.482 (-0.764 to -0.199)	0.001	0.474
**Peripheral neuropathy**					
None	Ref.		Ref.		
Mild	0.077 (-0.234 to 0.388)	0.628	0.195 (-0.092 to 0.481)	0.183	0.586
Moderate	-0.292 (-0.661 to 0.078)	0.122	-0.183 (-0.476 to 0.110)	0.220	0.652
**Fracture**					
None	Ref.		Ref.		
Moderate	-0.362 (-0.678 to -0.046)	0.025	-0.362 (-0.645 to -0.080)	0.012	0.999
Severe	-1.877 (-2.332 to -1.422)	<0.001	-1.312 (-1.694 to -0.930)	<0.001	0.063
**Ischemic heart disease**					
None	Ref.		Ref.		

Moderate	-0.807 (-1.150 to -0.463)	<0.001	-0.439 (-0.734 to -0.143)	0.004	0.111
Severe	-2.478 (-2.999 to -1.958)	<0.001	-2.039 (-2.442 to -1.637)	<0.001	0.191
Very severe	-3.859 (-4.502 to -3.216)	<0.001	-3.291 (-3.813 to -2.769)	<0.001	0.179
**Rash**					
None	Ref.		Ref.		
Mild	-0.459 (-0.682 to -0.235)	<0.001	-0.184 (-0.407 to 0.038)	0.104	0.088
Moderate	-0.712 (-1.036 to -0.388)	<0.001	-0.519 (-0.814 to -0.224)	0.001	0.390
**Standard deviations (SD)[b]**					
1-year OS benefit	1.496 (1.058 to 1.934)	<0.001	1.296 (0.919 to 1.674)	<0.001	0.498
**Model details**					
Log-likelihood	-482.3		-522.8		
AIC	994.6		1075.5		
BIC	1068.3		1149.5		

Legend: AIC: Akaike information criterion, BIC: Bayesian information criterion, Coeff.: coefficient (preference weight), CI: confidence interval, OS: overall survival, Ref.: reference. [a] P-values calculated using z-tests for a difference between prostate cancer and general population subsample participants, estimated based on separate mixed multinomial logit models. [b] Standard deviations derived from mixed multinomial logit models are a measure for the preference heterogeneity between individuals in the sample.

Subgroup Analyses

Supplementary Table 13: Preference weights (and 95% confidence intervals) derived from subgroup analyses among metastatic prostate cancer patients, stratified by age groups, disease stage, and experiences with adverse effects of treatment.

**Prostate cancer patients**	**Age**				**Disease stage**				**Adverse effects experience**			
	**45–64 years** (N=18)		**65+ years** (N=59)		**mHSPC** (N=57)		**mCRPC** (N=20)		**No experience** (N=22)		**Experienced** (N=49)	
	Coeff. (95% CI)	p-value	Coeff. (95% CI)	p-value	Coeff. (95% CI)	p-value	Coeff. (95% CI)	p-value	Coeff. (95% CI)	p-value	Coeff. (95% CI)	p-value
**Overall survival**												
1-year OS benefit	1.612 (0.412 to 2.811)	0.008	1.126 (0.726 to 1.527)	<0.001	1.322 (0.895 to 1.750)	<0.001	0.993 (0.030 to 1.957)	0.043	2.140 (1.026 to 3.253)	<0.001	1.029 (0.565 to 1.492)	<0.001
Test for difference between subgroups[a]				*0.452*				*0.541*				*0.071*
**Diarrhea**												
None	Ref.		Ref.		Ref.		Ref.		Ref.		Ref.	
Mild	-0.174 (-0.708 to 0.360)	0.523	-0.402 (-0.700 to -0.104)	0.008	-0.295 (-0.624 to 0.033)	0.078	-0.593 (-1.063 to -0.124)	0.013	-0.572 (-1.335 to 0.192)	0.142	-0.356 (-0.661 to -0.051)	0.022
Moderate	-0.623 (-1.204 to -0.042)	0.035	-0.753 (-1.036 to -0.470)	<0.001	-0.689 (-0.989 to -0.389)	<0.001	-0.873 (-1.375 to -0.371)	0.001	-0.690 (-1.287 to -0.093)	0.023	-0.771 (-1.077 to -0.465)	<0.001
**Fatigue**												
None	Ref.		Ref.		Ref.		Ref.		Ref.		Ref.	
Mild	-0.025 (-0.593 to 0.542)	0.930	-0.092 (-0.393 to 0.210)	0.551	-0.073 (-0.402 to 0.256)	0.662	-0.016 (-0.519 to 0.486)	0.949	0.184 (-0.708 to 1.075)	0.686	-0.135 (-0.441 to 0.170)	0.386

Moderate	-0.479 (-1.183 to 0.225)	0.183	-0.658 (-0.981 to -0.336)	<0.001	-0.631 (-0.975 to -0.288)	<0.001	-0.511 (-1.064 to 0.043)	0.071	-0.711 (-1.423 to 0.001)	0.050	-0.612 (-0.961 to -0.263)	0.001
**Peripheral neuropathy**												
None	Ref.		Ref.		Ref.		Ref.		Ref.		Ref.	
Mild	0.260 (-0.340 to 0.860)	0.396	-0.005 (-0.326 to 0.317)	0.978	0.262 (-0.083 to 0.607)	0.137	-0.512 (-1.059 to 0.036)	0.067	0.389 (-0.486 to 1.264)	0.383	-0.098 (-0.426 to 0.231)	0.559
Moderate	-0.423 (-1.142 to 0.296)	0.249	-0.214 (-0.591 to 0.163)	0.266	-0.145 (-0.566 to 0.276)	0.500	-0.483 (-1.071 to 0.106)	0.108	-0.582 (-1.820 to 0.656)	0.357	-0.248 (-0.618 to 0.123)	0.191
**Fracture**												
None	Ref.		Ref.		Ref.		Ref.		Ref.		Ref.	
Moderate	-0.492 (-1.169 to 0.184)	0.154	-0.286 (-0.604 to 0.031)	0.077	-0.137 (-0.483 to 0.208)	0.436	-0.689 (-1.220 to -0.158)	0.011	-0.295 (-0.942 to 0.352)	0.372	-0.349 (-0.691 to -0.007)	0.045
Severe	-1.451 (-2.321 to -0.580)	0.001	-1.864 (-2.319 to -1.408)	<0.001	-1.642 (-2.119 to -1.164)	<0.001	-2.197 (-2.995 to -1.399)	<0.001	-2.659 (-3.840 to -1.478)	<0.001	-1.622 (-2.090 to -1.154)	<0.001
**Ischemic heart disease**												
None	Ref.		Ref.		Ref.		Ref.		Ref.		Ref.	
Moderate	-0.244 (-0.866 to 0.378)	0.442	-0.854 (-1.210 to -0.498)	<0.001	-0.823 (-1.210 to -0.435)	<0.001	-0.504 (-1.055 to 0.047)	0.073	-1.339 (-2.182 to -0.497)	0.002	-0.517 (-0.869 to -0.164)	0.004
Severe	-1.818 (-2.817 to -0.818)	<0.001	-2.471 (-2.992 to -1.951)	<0.001	-2.496 (-3.060 to -1.931)	<0.001	-2.010 (-2.839 to -1.181)	<0.001	-3.333 (-4.913 to -1.753)	<0.001	-2.069 (-2.574 to -1.564)	<0.001

Very severe	-3.332 (-4.588 to -2.075)	<0.001	-3.745 (-4.382 to -3.109)	<0.001	-3.813 (-4.491 to -3.136)	<0.001	-3.447 (-4.535 to -2.359)	<0.001	-4.515 (-6.289 to -2.741)	<0.001	-3.484 (-4.131 to -2.837)	<0.001
**Rash**												
None	Ref.		Ref.		Ref.		Ref.		Ref.		Ref.	
Mild	-0.070 (-0.509 to 0.369)	0.754	-0.525 (-0.753 to -0.297)	<0.001	-0.492 (-0.726 to -0.258)	<0.001	-0.319 (-0.718 to 0.079)	0.116	-0.566 (-0.998 to -0.134)	0.010	-0.402 (-0.647 to -0.157)	0.001
Moderate	-0.380 (-1.000 to 0.240)	0.230	-0.745 (-1.080 to -0.409)	<0.001	-0.683 (-1.043 to -0.323)	<0.001	-0.671 (-1.233 to -0.110)	0.019	-0.843 (-1.634 to -0.052)	0.037	-0.629 (-0.979 to -0.280)	<0.001
**Standard deviations (SD)[b]**												
1-year OS benefit	2.127 (0.924 to 3.330)	0.001	1.097 (0.722 to 1.473)	<0.001	1.049 (0.667 to 1.431)	<0.001	1.950 (0.983 to 2.917)	<0.001	1.657 (0.750 to 2.563)	<0.001	1.258 (0.810 to 1.705)	<0.001
**Model details**												
Log-likelihood	-132.3		-441.0		-406.7		-162.0		-140.9		-387.0	
AIC	294.6		911.9		843.4		354.1		311.9		803.9	
BIC	348.8		984.2		914.9		410.6		368.5		873.6	

Legend: AIC: Akaike information criterion, BIC: Bayesian information criterion, Coeff.: coefficient (preference weight), CI: confidence interval, OS: overall survival, Ref.: reference. [a] P-values calculated using z-tests for a difference between subgroup participants, estimated based on separate mixed multinomial logit models. [b] Standard deviations derived from mixed multinomial logit models are a measure for the preference heterogeneity between individuals in the sample.

Supplementary Table 14: Preference weights (and 95% confidence intervals) derived from subgroup analyses among men from the general population, stratified by age groups, and personal or professional experiences with cancer.

Men from general population	Age	Personal or professional experiences with cancer

	45–64 years (N=156)		65+ years (N=157)		No experience (N=74)		Experienced (N=236)	
	Coeff. (95% CI)	p-value	Coeff. (95% CI)	p-value	Coeff. (95% CI)	p-value	Coeff. (95% CI)	p-value
**Overall survival**								
1-year OS benefit	0.633 (0.433 to 0.834)	<0.001	0.526 (0.301 to 0.750)	<0.001	0.640 (0.341 to 0.940)	<0.001	0.569 (0.394 to 0.744)	<0.001
Test for difference between subgroups[a]				*0.483*				*0.688*
**Diarrhea**								
None	Ref.		Ref.		Ref.		Ref.	
Mild	-0.088 (-0.255 to 0.080)	0.306	-0.256 (-0.424 to -0.089)	0.003	-0.251 (-0.483 to -0.019)	0.034	-0.156 (-0.293 to -0.018)	0.026
Moderate	-0.616 (-0.776 to -0.457)	<0.001	-0.593 (-0.754 to -0.433)	<0.001	-0.561 (-0.800 to -0.322)	<0.001	-0.626 (-0.755 to -0.496)	<0.001
**Fatigue**								
None	Ref.		Ref.		Ref.		Ref.	
Mild	-0.164 (-0.326 to -0.002)	0.048	-0.240 (-0.402 to -0.077)	0.004	-0.231 (-0.465 to 0.003)	0.053	-0.193 (-0.325 to -0.061)	0.004
Moderate	-0.751 (-0.938 to -0.565)	<0.001	-0.750 (-0.937 to -0.563)	<0.001	-0.966 (-1.249 to -0.684)	<0.001	-0.689 (-0.839 to -0.539)	<0.001
**Peripheral neuropathy**								
None	Ref.		Ref.		Ref.		Ref.	
Mild	0.089 (-0.089 to 0.267)	0.328	0.093 (-0.084 to 0.270)	0.304	-0.037 (-0.288 to 0.213)	0.770	0.140 (-0.005 to 0.285)	0.059
Moderate	-0.100 (-0.294 to 0.094)	0.312	-0.262 (-0.453 to -0.072)	0.007	-0.275 (-0.546 to -0.005)	0.046	-0.155 (-0.312 to 0.002)	0.053
**Fracture**								
None	Ref.		Ref.		Ref.		Ref.	
Moderate	-0.439 (-0.621 to -0.258)	<0.001	-0.437 (-0.617 to -0.257)	<0.001	-0.579 (-0.845 to -0.312)	<0.001	-0.400 (-0.547 to -0.253)	<0.001
Severe	-1.659 (-1.907 to -1.412)	<0.001	-1.444 (-1.690 to -1.198)	<0.001	-1.637 (-1.992 to -1.282)	<0.001	-1.529 (-1.730 to -1.327)	<0.001
**Ischemic heart disease**								
None	Ref.		Ref.		Ref.		Ref.	
Moderate	-0.821 (-1.016 to -0.626)	<0.001	-0.495 (-0.680 to -0.310)	<0.001	-0.595 (-0.860 to -0.331)	<0.001	-0.671 (-0.826 to -0.515)	<0.001
Severe	-2.268 (-2.535 to -2.001)	<0.001	-1.957 (-2.213 to -1.702)	<0.001	-1.887 (-2.255 to -1.518)	<0.001	-2.189 (-2.404 to -1.975)	<0.001

Very severe	-3.430 (-3.765 to -3.094)	<0.001	-3.301 (-3.634 to -2.967)	<0.001	-3.312 (-3.791 to -2.832)	<0.001	-3.395 (-3.669 to -3.121)	<0.001
**Rash**								
None	Ref.		Ref.		Ref.		Ref.	
Mild	-0.272 (-0.411 to -0.133)	<0.001	-0.191 (-0.330 to -0.051)	0.007	-0.349 (-0.550 to -0.148)	0.001	-0.199 (-0.312 to -0.085)	0.001
Moderate	-0.618 (-0.804 to -0.432)	<0.001	-0.404 (-0.589 to -0.219)	<0.001	-0.440 (-0.705 to -0.175)	0.001	-0.540 (-0.691 to -0.388)	<0.001
**Standard deviations (SD)[b]**								
1-year OS benefit	0.928 (0.734 to 1.123)	<0.001	1.129 (0.913 to 1.346)	<0.001	0.969 (0.678 to 1.261)	<0.001	1.076 (0.904 to 1.248)	<0.001
**Model details**								
Log-likelihood	-1253.3		-1281.3		-611.3		-1917.5	
AIC	2536.6		2592.5		1252.5		3865.0	
BIC	2623.9		2679.8		1328.7		3958.5	

Legend: AIC: Akaike information criterion, BIC: Bayesian information criterion, Coeff.: coefficient (preference weight), CI: confidence interval, OS: overall survival, Ref.: reference. [a] P-values calculated using z-tests for a difference between subgroup participants, estimated based on separate mixed multinomial logit models. [b] Standard deviations derived from mixed multinomial logit models are a measure for the preference heterogeneity between individuals in the sample.

Latent Class Analysis

Supplementary Table 15: Preference weights derived based on latent class multinomial logit models assessing the presence of two groups with different sets of preferences among study participants (overall study population, N=388).

	**Class 1** (N=295)		**Class 2** (N=93)		**Test for difference**[a]
	Coeff. (95% CI)	p-value	Coeff. (95% CI)	p-value	p-value
**Overall survival**					
1-year OS benefit	0.204 (0.100 to 0.308)	<0.001	1.488 (1.093 to 1.883)	<0.001	<0.001
**Diarrhea**					
None	Ref.		Ref.		
Mild	-0.166 (-0.305 to -0.027)	0.019	-0.169 (-0.410 to 0.072)	0.170	0.982
Moderate	-0.616 (-0.739 to -0.494)	<0.001	-0.279 (-0.552 to -0.005)	0.046	0.027
**Fatigue**					
None	Ref.		Ref.		
Mild	-0.051 (-0.178 to 0.075)	0.427	-0.180 (-0.421 to 0.062)	0.145	0.356
Moderate	-0.599 (-0.732 to -0.466)	<0.001	-0.526 (-0.838 to -0.214)	0.001	0.676
**Peripheral neuropathy**					
None	Ref.		Ref.		
Mild	0.073 (-0.072 to 0.219)	0.325	-0.019 (-0.301 to 0.263)	0.897	0.571
Moderate	0.003 (-0.137 to 0.142)	0.972	-0.042 (-0.309 to 0.224)	0.756	0.770
**Fracture**					
None	Ref.		Ref.		
Moderate	-0.372 (-0.506 to -0.239)	<0.001	-0.118 (-0.424 to 0.189)	0.451	0.136
Severe	-1.493 (-1.695 to -1.292)	<0.001	-0.579 (-0.896 to -0.262)	<0.001	<0.001

**Ischemic heart disease**					
None	Ref.		Ref.		
Moderate	-0.634 (-0.783 to -0.485)	<0.001	-0.062 (-0.331 to 0.206)	0.649	<0.001
Severe	-2.296 (-2.562 to -2.029)	<0.001	-0.243 (-0.685 to 0.198)	0.280	<0.001
Very severe	-3.531 (-3.889 to -3.173)	<0.001	-1.304 (-1.763 to -0.846)	<0.001	<0.001
**Rash**					
None	Ref.		Ref.		
Mild	-0.268 (-0.387 to -0.148)	<0.001	-0.057 (-0.283 to 0.169)	0.621	0.106
Moderate	-0.528 (-0.688 to -0.368)	<0.001	-0.234 (-0.485 to 0.017)	0.067	0.053
**Model details**					
Log-likelihood	-3120.1				
AIC	6298.1				
BIC	6493.2				

Legend: AIC: Akaike information criterion, BIC: Bayesian information criterion, Coeff.: coefficient (preference weight), CI: confidence interval, OS: overall survival, Ref.: reference. [a] P-values calculated using z-tests for a difference between participants in the two classes, estimated based on coefficients derived from the latent class model.

References

[1] Medical Device Innovation Consortium (MDIC). Patient Centered Benefit-Risk Project Report: A Framework for Incorporating Information on Patient Preferences Regarding Benefit and Risk into Regulatory Assessments of New Medical Technology 2015. https://www.fda.gov/media/95591/download (accessed May 11, 2022).

[2] van Overbeeke E, Whichello C, Janssens R, Veldwijk J, Cleemput I, Simoens S, et al. Factors and situations influencing the value of patient preference studies along the medical product lifecycle: a literature review. Drug Discovery Today 2019;24:57–68. https://doi.org/10.1016/j.drudis.2018.09.015.

[3] Soekhai V, Whichello C, Levitan B, Veldwijk J, Pinto CA, Donkers B, et al. Methods for exploring and eliciting patient preferences in the medical product lifecycle: a literature review. Drug Discovery Today 2019;24:1324–31. https://doi.org/10.1016/j.drudis.2019.05.001.

[4] Whichello C, Bywall KS, Mauer J, Stephen W, Cleemput I, Pinto CA, et al. An overview of critical decision-points in the medical product lifecycle: Where to include patient preference information in the decision-making process? Health Policy 2020;124:1325–32. https://doi.org/10.1016/j.healthpol.2020.07.007.

[5] Bridges JFP, Hauber AB, Marshall D, Lloyd A, Prosser LA, Regier DA, et al. Conjoint Analysis Applications in Health—a Checklist: A Report of the ISPOR Good Research Practices for Conjoint Analysis Task Force. Value in Health 2011;14:403–13. https://doi.org/10.1016/j.jval.2010.11.013.

[6] Johnson FR, Lancsar E, Marshall D, Kilambi V, Mühlbacher A, Regier DA, et al. Constructing Experimental Designs for Discrete-Choice Experiments: Report of the ISPOR Conjoint Analysis Experimental Design Good Research Practices Task Force. Value in Health 2013;16:3–13. https://doi.org/10.1016/j.jval.2012.08.2223.

[7] Hauber AB, González JM, Groothuis-Oudshoorn CGM, Prior T, Marshall DA, Cunningham C, et al. Statistical Methods for the Analysis of Discrete Choice Experiments: A Report of the ISPOR Conjoint Analysis Good Research Practices Task Force. Value in Health 2016;19:300–15. https://doi.org/10.1016/j.jval.2016.04.004.

[8] Menges D, Piatti MC, Cerny T, Puhan MA. Patient Preference Studies for Advanced Prostate Cancer Treatment Along the Medical Product Life Cycle: Systematic Literature Review. PPA 2022;16:1539–57. https://doi.org/10.2147/PPA.S362802.

[9] Menges D, Braun J, Piatti MC, Puhan M. Patient Preferences Regarding Benefits and Harms of Advanced Prostate Cancer Treatments in Switzerland. Open Science Framework (OSF) Registrations 2021. https://doi.org/10.17605/OSF.IO/UN682 (accessed May 11, 2022).

[10] Gale NK, Heath G, Cameron E, Rashid S, Redwood S. Using the framework method for the analysis of qualitative data in multi-disciplinary health research. BMC Medical Research Methodology 2013;13:117. https://doi.org/10.1186/1471-2288-13-117.

[11] Doveson S, Holm M, Axelsson L, Fransson P, Wennman-Larsen A. Facing life-prolonging treatment: The perspectives of men with advanced metastatic prostate cancer – An interview study. European Journal of Oncology Nursing 2020;49:101859. https://doi.org/10.1016/j.ejon.2020.101859.

[12] de Freitas HM, Ito T, Hadi M, Al-Jassar G, Henry-Szatkowski M, Nafees B, et al. Patient Preferences for Metastatic Hormone-Sensitive Prostate Cancer Treatments: A Discrete Choice Experiment Among Men in Three European Countries. Adv Ther 2019;36:318–32. https://doi.org/10.1007/s12325-018-0861-3.

[13] Eliasson L, de Freitas HM, Dearden L, Calimlim B, Lloyd AJ. Patients’ Preferences for the Treatment of Metastatic Castrate-resistant Prostate Cancer: A Discrete Choice Experiment. Clinical Therapeutics 2017;39:723–37. https://doi.org/10.1016/j.clinthera.2017.02.009.

[14] Lloyd A, Penson D, Dewilde S, Kleinman L. Eliciting patient preferences for hormonal therapy options in the treatment of metastatic prostate cancer. Prostate Cancer and Prostatic Diseases 2008;11:153–9. https://doi.org/10.1038/sj.pcan.4500992.

[15] Uemura H, Matsubara N, Kimura G, Yamaguchi A, Ledesma DA, DiBonaventura M, et al. Patient preferences for treatment of castration-resistant prostate cancer in Japan: a discrete-choice experiment. BMC Urology 2016;16:63. https://doi.org/10.1186/s12894-016-0182-2.

[16] Nakayama M, Kobayashi H, Okazaki M, Imanaka K, Yoshizawa K, Mahlich J. Patient Preferences and Urologist Judgments on Prostate Cancer Therapy in Japan. Am J Mens Health 2018;12:1094–101. https://doi.org/10.1177/1557988318776123.

[17] Srinivas S, Mohamed AF, Appukkuttan S, Botteman M, Ng X, Joshi N, et al. Patient and caregiver benefit-risk preferences for nonmetastatic castration-resistant prostate cancer treatment. Cancer Med 2020;9:6586–96. https://doi.org/10.1002/cam4.3321.

[18] Menges D, Yebyo HG, Sivec-Muniz S, Haile SR, Barbier MC, Tomonaga Y, et al. Treatments for Metastatic Hormone-sensitive Prostate Cancer: Systematic Review, Network Meta-analysis, and Benefit-harm assessment. European Urology Oncology 2022;5:605–16. https://doi.org/10.1016/j.euo.2022.04.007.

[19] de Bekker-Grob EW, Ryan M, Gerard K. Discrete choice experiments in health economics: a review of the literature. Health Economics 2012;21:145–72. https://doi.org/10.1002/hec.1697.

[20] Harrison M, Rigby D, Vass C, Flynn T, Louviere J, Payne K. Risk as an Attribute in Discrete Choice Experiments: A Systematic Review of the Literature. Patient 2014;7:151–70. https://doi.org/10.1007/s40271-014-0048-1.

[21] Vass CM, Payne K. Using Discrete Choice Experiments to Inform the Benefit-Risk Assessment of Medicines: Are We Ready Yet? Pharmacoeconomics 2017;35:859–66. https://doi.org/10.1007/s40273-017-0518-0.

[22] Mühlbacher AC, Bethge S, Sadler A. Compound Attributes For Side Effect In Discrete Choice Experiments: Risk or Severity - What Is More Important to Hepatitis C Patients? Value in Health 2015;18:A629–30. https://doi.org/10.1016/j.jval.2015.09.2223.

[23] United States Department of Health and Human Services, National Cancer Institute (NCI). Common Terminology Criteria for Adverse Events (CTCAE) 2020. https://ctep.cancer.gov/protocoldevelopment/electronic_applications/ctc.htm (accessed October 23, 2021).

[24] Barbier MC, Tomonaga Y, Menges D, Yebyo HG, Haile SR, Puhan MA, et al. Survival modelling and cost-effectiveness analysis of treatments for newly diagnosed metastatic hormone-sensitive prostate cancer. PLOS ONE 2022;17:e0277282. https://doi.org/10.1371/journal.pone.0277282.

[25] R Core Team. R: A language and environment for statistical computing. R Foundation for Statistical Computing. Vienna, Austria 2017.

[26] Traets F, Sanchez DG, Vandebroek M. Generating Optimal Designs for Discrete Choice Experiments in R: The idefix Package. Journal of Statistical Software 2020;96:1–41. https://doi.org/10.18637/jss.v096.i03.

[27] Faeh D, Minder C, Gutzwiller F, Bopp M, Group for the SNCS. Culture, risk factors and mortality: can Switzerland add missing pieces to the European puzzle? Journal of Epidemiology & Community Health 2009;63:639–45. https://doi.org/10.1136/jech.2008.081042.

[28] Hurst SA, Zellweger U, Bosshard G, Bopp M, Faisst K, Gutzwiller F, et al. Medical end-of-life practices in Swiss cultural regions: a death certificate study. BMC Medicine 2018;16:54. https://doi.org/10.1186/s12916-018-1043-5.

[29] de Bekker-Grob EW, Donkers B, Jonker MF, Stolk EA. Sample Size Requirements for Discrete-Choice Experiments in Healthcare: a Practical Guide. Patient 2015;8:373–84. https://doi.org/10.1007/s40271-015-0118-z.

[30] Orme BK. Getting started with conjoint analysis: strategies for product design and pricing research. Research Publishers, LLC Madison, WI; 2006.
